# The cell cycle regulator PLK1 promotes murine melanoma progression by regulating the transcription factor BACH1

**DOI:** 10.1371/journal.pbio.3003490

**Published:** 2025-11-24

**Authors:** Fengyi Mao, Sai Wu, Derek B. Allison, Daheng He, Yifan Kong, Chaohao Li, Zhiguo Li, Yanquan Zhang, Xinyi Wang, Qiongsi Zhang, Chi Wang, Xiaoqi Liu

**Affiliations:** 1 Department of Toxicology and Cancer Biology, University of Kentucky, Lexington, Kentucky, United States of America; 2 Markey Cancer Center, University of Kentucky, Lexington, Kentucky, United States of America; 3 Department of Pathology and Laboratory Medicine, University of Kentucky, Lexington, Kentucky, United States of America; The Institute of Cancer Research, UNITED KINGDOM OF GREAT BRITAIN AND NORTHERN IRELAND

## Abstract

Polo-like kinase 1 (PLK1), a critical cell cycle regulator, is associated with cancer progression and negatively correlates with patient survival in cutaneous melanoma based on clinical database analysis. In a melanoma mouse model induced by *BRaf*^*CA*^ mutation and *Pten*-deficiency, we observed that PLK1 overexpression mediated metabolic reprogramming to markedly accelerate tumor growth, promote metastasis, and shortened mice survival. Mechanistically, PLK1 stabilizes BTB domain and CNC homolog 1 (BACH1), which serves as a crucial transcription factor for genes involved in cancer metabolism and metastasis. Moreover, the PLK1/BACH1 axis confers resistance to Vemurafenib, a BRAF^V600E^ inhibitor, in melanoma. In light of this finding, we attempted an innovative pharmacological combination targeting both BRAF^V600E^ and PLK1, identifying a synergistic efficiency to this approach to suppress tumor growth. Overall, we have discovered a novel function of PLK1 that is independent of the cell cycle, which could pave new ways for melanoma therapies.

## Abstract 2

The cell cycle regulator PLK1 is associated with cancer progression and correlates with patient survival in cutaneous melanoma, although the mechanisms remain unclear. This study uses a mouse model of melanoma to show that PLK1 stabilizes BACH1, which transcriptionally regulates genes involved in cancer metabolism and metastasis, independently of the cell cycle.

## Introduction

In the United States, cutaneous melanoma is one of the most common and fatal types of skin cancer among Whites. It is estimated that there will be 104,960 new cases and 8,430 deaths in 2025 [1]. Malignant melanoma develops from melanocytes that have accumulated sustained genetic mutations and alterations [2]. Whole genome analyses have revealed that melanoma typically carries a high load of gene mutations (>10 mutations per megabase), with a signature that is typical of ultraviolet-induced mutations [3]. More specifically, over 50% of patients with advanced melanoma harbor activating mutations in the BRAF oncogene [3,4]. The V600E point mutation is the most observed BRAF alteration among patients and leads to a significant increase in kinase activity, resulting in constitutive activation of the mitogen-activated protein kinase (MAPK) signaling pathway [5]. Vemurafenib (PLX-4032) was approved by the FDA in 2011 for the treatment of metastatic malignant melanoma by specifically targeting the BRAF^V600E^ mutation [6,7]. However, the overall duration of response to Vemurafenib is relatively limited, lasting only about 6 months [6,7]. This highlights the urgent need for novel therapies in the treatment of melanoma.

Although an activating mutation in the BRAF protein is often present, the malignant transformation from benign nevus to deadly melanoma requires dysregulation in other classic molecular pathways, such as the RB1/CDKN2A cell cycle pathway, the MDM2/TP53 apoptosis pathway, and the PI3K-AKT cell survival pathway [4]. Interestingly, PTEN deletions and mutations were found to be more frequent when accompanied by BRAF mutations, suggesting cooperation between BRAF and PTEN in tumorigenesis [4]. Furthermore, this correlation was also observed with the progression of melanoma metastasis [4,8]. Metastatic melanoma has a poor 5-year overall survival rate, making it highly lethal [2]. Metabolic adaptation is a prerequisite for the successful metastasis of cancer cells to lymph nodes and other distant organs [9]. Several recent publications have shown that invasive melanoma cells preferentially rely on glycolysis for energy supply. In addition, the high frequency of metastasis in melanoma has been linked to lower intracellular levels of reactive oxygen species (ROS), increased capacity to counteract oxidative stress, and suppression of mitochondrial energetic metabolism [10–12].

Polo-like kinase 1 (PLK1) is a well-known regulator of the cell cycle and participates in multiple mitotic processes, thus gaining considerable interest as a novel target in cancer treatment [13]. Numerous publications reveal PLK1’s role in promoting cancer development, progression, and resistance to chemotherapy agents, such as doxorubicin, gemcitabine, and taxol, in multiple types of cancers [14]. Importantly, PLK1 is known to activate the PI3K/PTEN and the CRAF/MEK signaling pathways, which subsequently lead to aerobic glycolysis and epithelial-mesenchymal transition (EMT) [15,16]. As a result, numerous studies have demonstrated the potential of PLK1 as a potent target for cancer treatment, and clinical trials are currently underway to validate the efficacy and safety profiles of PLK1 inhibitors [14]. Volasertib (BI6727) is among the most promising inhibitors, being a highly potent ATP-competitive inhibitor of PLK1 [17]. Studies have validated its efficacy in impeding cell proliferation, inducing cell cycle arrest, and promoting cell death [18,19]. Additionally, it has been reported that PLK1 inhibitor could synergize with MEK inhibitor in NRAS mutant melanoma [20]. Despite the aforementioned progress, whether PLK1 drives melanoma progression requires further investigation in vitro and in vivo. Herein, we investigated the role of PLK1 in melanoma progression using the genetically engineered mouse (GEM) model *Braf*^*CA/+*^ */ Pten*^*loxp/loxp*^ */ Tyr::CreER*^*T2*^ [21,22]. In this study, we found that PLK1 regulates melanoma’s metabolic reprogramming, metastasis, and drug resistance by stabilizing BTB domain and CNC homolog 1 (BACH1). Notably, our results demonstrate that combining Volasertib, a highly potent ATP-competitive inhibitor of PLK1, with the current standard of care drug, Vemurafenib, robustly enhances its efficacy. This finding highlights PLK1 as an effective alternative to overcome drug resistance and treat melanoma patients. Overall, the present study highlights the critical role of PLK1 in melanoma progression and demonstrates the potential of PLK1 inhibitors, such as Volasertib, as a promising treatment option for patients with melanoma.

## Results

### PLK1 overexpression promotes melanoma development and progression

**Fig 1:**
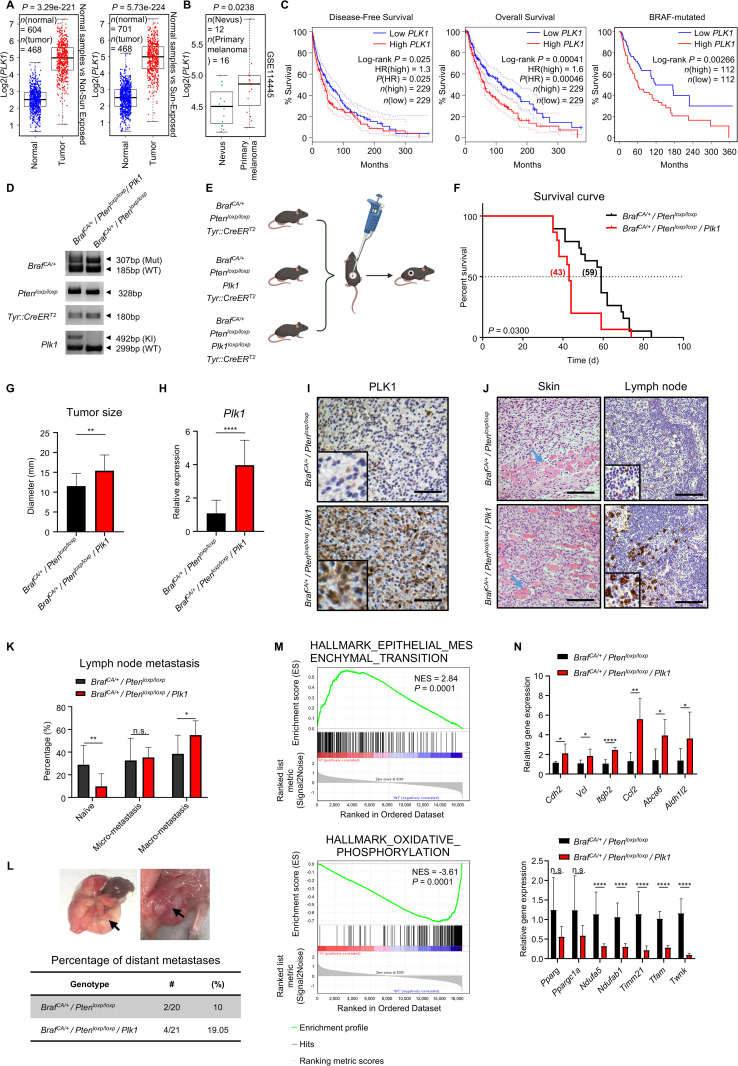
Polo-like kinase 1 (PLK1) is involved in melanoma progression. **(A)** *PLK1* expression in melanoma tumors compared to the normal tissues from The Cancer Genome Atlas database. Left, non-sun-exposed melanoma. Right, sun-exposed melanoma. *P* value by unpaired Student *t* test. **(B)** *PLK1* expression in primary melanoma tumors vs. nevus in dataset GSE114445. *P* value by unpaired Student *t* test. **(C)** Expression of *PLK1* negatively correlates to human melanoma patient survival. Left, disease-free survival. Middle, overall survival. Right, patients harboring BRAF mutations. The number (*n*) of patients in each group is indicated in the graph respectively. The patients were dichotomized into low and high groups based on the median expression level of *PLK1*. Log-rank (Mantel–Cox) test. HR, hazard rate ratio. **(D)** A representative image of indicated mouse genotypes confirmed by PCR analysis. **(E)** Schematic showing the localized induction of melanoma in the *Braf*^*CA/+*^ */ Pten*^*loxp/loxp*^ mouse model. 4-OHT, 4-Hydroxytamoxifen. The figure was generated with BioRender.com. **(F)** Survival curve of *Braf*^*CA/+*^ */ Pten*^*loxp/loxp*^ */ Plk1* mice vs. *Braf*^*CA/+*^ */ Pten*^*loxp/loxp*^ mice. *n* = 15 and 19, respectively. The mice were euthanized when the average diameter of tumors reached 15 mm. Log-rank (Mantel–Cox) test. **(G)** Tumor diameter after 40 d of localized induction. Mean ± SD. * *P* < 0.05; ** *P* < 0.01 by unpaired Student *t* tes*t*. *n* = 20 and 13, respectively. (**H**) mRNA level of *Plk1* in melanoma tumors of the indicated genotypes. Mean ± SD. * *P* < 0.05; ** *P* < 0.01; *** *P* < 0.001; **** *P* < 0.0001 by unpaired Student *t* test. *n* = 5 biological replicates. **(I)** Representative IHC staining for PLK1 in the locally induced melanoma tumors. Scale bar, 150 μm. **(J)** H&E staining of tissues collected from mice with indicated genotypes. Left, melanoma tissues. The blue arrow points to the muscle layer. Right, tumor-draining lymph nodes. Black dots are metastasized melanoma cells expressing melanin. Scale bar, 150 μm. **(K)** Quantification of metastasis to lymph nodes in *Braf*^*CA/+*^ */ Pten*^*loxp/loxp*^ and *Braf*^*CA/+*^ */ Pten*^*loxp/loxp*^ */ Plk1* mice. The mice were euthanized when the average diameter of tumors reached 15 mm. Mean ± SD. * *P* < 0.05; ** *P* < 0.01 by unpaired Student *t* test. *n* = 13 and 12, respectively, for *Braf*^*CA/+*^ */ Pten*^*loxp/loxp*^ and *Braf*^*CA/+*^ */ Pten*^*loxp/loxp*^*/ Plk1* mice. **(L)** Representative images and quan*t*ification of distant metastasis found in indicated mice. **(M)** GSEA correlation of EPITHELIAL_MESENCHYMAL_TRANSITION (Top) and OXIDATIVE_PHOSPHORYLATION (Bottom) with alternatively expressed genes in tumors of *Braf*^*CA/+*^ */ Pten*^*loxp/loxp*^ */ Plk1* vs. *Braf*^*CA/+*^ */ Pten*^*loxp/loxp*^. Normalized enrichment scores and nominal *P* values are shown. **(N)** The mRNA expression level of indicated genes in the mouse melanoma tumors. Mean ± SD. *n* = 5 biological replicates. n.s., *P* > 0.05; * *P* < 0.05; ** *P* < 0.01; *** *P* < 0.001; **** *P* < 0.0001 by unpaired Student *t* test. The data underlying the graphs shown in the figure can be found in S1 Data.

To investigate the role of PLK1 in melanoma, we first examined its clinical relevance using The Cancer Genome Atlas (TCGA) database. We found that compared to normal skin, the expression of *PLK1* was significantly increased in both non-sun-exposed and sun-exposed melanoma (Fig 1A). Besides, a robust elevation of *PLK1* expression was observed in primary melanoma tissues, in comparison to the benign melanocytic nevus (Fig 1B). Notably, patients with high *PLK1* mRNA levels exhibited a significant reduction in disease-free and overall survival when compared to those with low *PLK1* expression (Fig 1C). Since BRAF is the most abundant driver mutation in melanoma [3], we also analyzed the *PLK1* expression in patients with melanoma harboring BRAF mutations. Consistent with previous studies [23], our results demonstrated that higher PLK1 expression was associated with worse survival outcomes in these patients (Fig 1C). *PLK1* expression level did not differ significantly between patients harboring wild-type or mutant BRAF, and patients with high *PLK1* expression showed a markedly increased overall *PLK1* level (S1A and S1B Fig). Taken together, our findings pointed out that PLK1 may contribute to melanoma progression and be correlated with poorer patient survival.

The conditional expression of BRAF^V600E^ mutation, along with *Pten* silencing in melanocytes, could robustly elicit melanoma progression with high penetrance, short latency, and apparent metastasis in mice, which makes it a valuable model system to study melanoma development [21,22]. To investigate the role of PLK1, we utilized the *Braf*^*CA/+*^ */ Pten*^*loxp/loxp*^ */ Tyr::CreER*^*T2*^ GEM model in vivo and incorporated either *Plk1* [24] or *Plk1*^*loxp/loxp*^ transgenes [25], to modulate the expression level of *Plk1*. Upon successful validation of the mice genotypes (Fig 1D), 4-Hydroxy-Tamoxifen (4-OHT) was then applied to the skin to introduce the expression of the transgenes, resulting in the formation of localized melanoma tumors (Fig 1E). To investigate the impact of *Plk1* on mouse melanoma development, we monitored the tumor growth for both long-term overall survival and at fixed time points. Consistent with our observations in human patients, *Plk1* overexpression significantly shortened survival, reducing the median survival from 59 d to 43 d compared to *Braf*^*CA/+*^ */ Pten*^*loxp/loxp*^ mice (Fig 1F). Conversely, *Plk1* depletion markedly delayed tumor formation and progression. Although *Braf*^*CA/+*^ */ Pten*^*loxp/loxp*^ */ Plk1*^*loxp/loxp*^ mice eventually developed melanoma, their median survival was prolonged to 106.5 d with a much slower proliferative rate (S1C Fig). However, Plk1 protein was still detectable in tumors derived from *Plk1*-depleted mice (S1C Fig), suggesting the incomplete *Plk1* depletion due to leaky Cre recombinase activity may have contributed to sustained tumor growth. Intriguingly, depletion of one-allele *Plk1* showed no impact on overall survival (S1C Fig). Consistently, the average diameter of the induced tumors was much vaster upon *Plk1* overexpression post 40 days of induction (Fig 1G). Hereafter, we validated *Plk1* overexpression in mouse tumors at mRNA and protein levels (Figs 1H, 1I, and S1I). The *Plk1* mRNA expression level was markedly increased, approximately 4-fold, in *Braf*^*CA/+*^ */ Pten*^*loxp/loxp*^ */ Plk1* mice. This increase was comparable to the observed elevation of *PLK1* in the melanoma patients with low and high PLK1 expression (S1B Fig). Of note, ample evidence indicates that elevated *Plk1* expression promotes metastasis in mouse melanoma, characterized by the migration of numerous cells through muscular tissue and the increased presence of pigmented metastatic sites in the draining lymph nodes (Figs 1J and S1D). In addition, the frequency of lymph nodes carrying macro-metastasis increased in *Braf*^*CA/+*^ */ Pten*^*loxp/loxp*^ */ Plk1* mice compared to *Braf*^*CA/+*^ */ Pten*^*loxp/loxp*^ mice (Figs 1K and S1E), while the incidence of distant metastasis was raised but did not reach statistical significance (Fig 1L).

To further explore the underlying role of *Plk1* in melanoma development, we performed RNA sequencing (RNA-seq) on mouse melanoma tumors. We then queried the RNA-seq results for “Hallmark” gene signatures from MSigDB [26] to identify the major pathways affected by high expression of *Plk1*. Based on the gene set enrichment analysis (GSEA), we identified that the EPITHELIAL_MESENCHYMAL_TRANSITION and KRAS_SIGNALING_UP pathways were highly enriched in the *Plk1* overexpressed tumors (Figs 1M, S1F, and S1G). These findings were consistent with the increased metastasis we observed in the mice. Interestingly, OXIDATIVE_PHOSPHORYLATION and OXYGEN_SPECIES_PATHWAY were dramatically repressed among all the cancer hallmark signatures. Similarly, the KEGG pathway enrichment analysis also indicated that oxidative phosphorylation (OXPHOS) was the most altered pathway upon *Plk1* overexpression (S1H Fig). To validate the results of RNA-seq, the mRNA level of the top-regulated genes was assessed by quantitative real-time PCR (qRT-PCR) (Fig 1N). In agreement, the metastatic-related *Cdh2* and *Vcl* were significantly upregulated, while the expression of mitochondrial-related genes was deeply repressed in the tumors of *Braf*^*CA/+*^ */ Pten*^*loxp/loxp*^ */ Plk1*. Immunoblot analysis showed varied expression of Vimentin among different tumors, while the key metastatic marker N-Cadherin were upregulated with overexpressed *Plk1* (S1I Fig). Besides, reduced expression of mitochondrial-related proteins had been observed, consistent with mRNA levels. Additionally, the antioxidant proteins, SOD2, Catalases, and NADPH Oxidase NOX1 exhibited a decreased trend in the *Plk1* overexpressed tumors, indicating the tumors may maintain a lower ROS status. Overall, these findings suggested that high expression of *Plk1* contributes to the melanoma growth, metabolic adaptation, and metabolism.

### PLK1 overexpression contributes to melanoma metastasis

**Fig 2:**
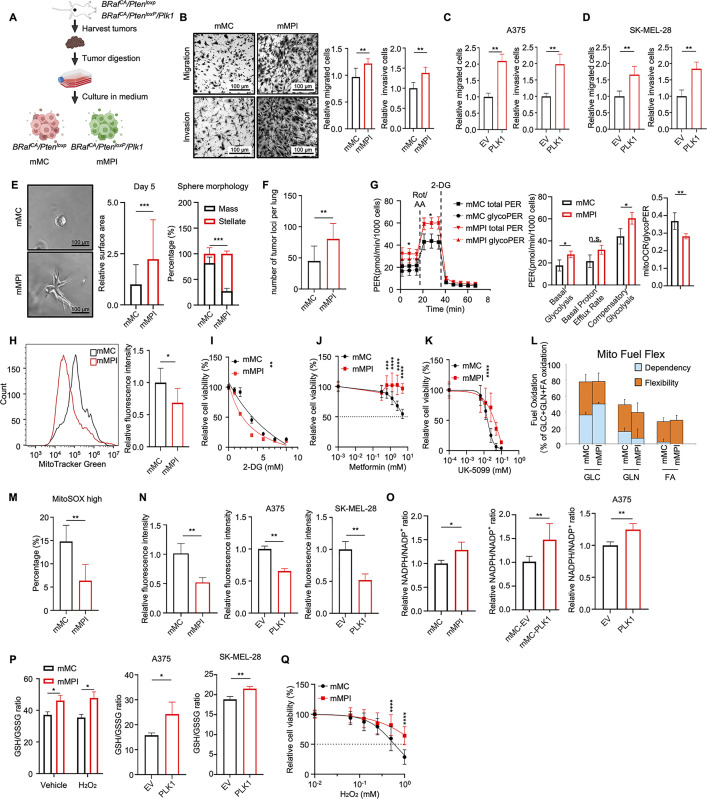
Overexpressed Polo-like kinase 1 (PLK1) promotes metastasis and metabolic reprogramming in melanoma. **(A)** Schematic showing the generation of stable mouse melanoma cells from *Braf*^*CA/+*^ */ Pten*^*loxp/loxp*^ to *Braf*^*CA/+*^ */ Pten*^*loxp/loxp*^ */ Plk1* mice, as mouse melanoma cells (mMC) and mouse melanoma with Plk1 knock-in (mMPI), respectively. The figure was generated with BioRender.com. **(B)** Transwell assay of mMC and mMPI cells. Left, representative images of migration assay (top) and invasion assay (bottom). Scale bar, 100 μm. *n* = 3 biological replicates. Right, quantification of transwell assay. Mean ± SD. * *P* < 0.05; ** *P* < 0.01; *** *P* < 0.001 by unpaired Student *t* test. **(C and D)** Quantification of A375 (C) and SK-MEL-28 (D) transwell assay. Left, migration assay. Right, invasion assay. Mean ± SD. * *P* < 0.05; ** *P* < 0.01; *** *P* < 0.001 by unpaired Student *t* test. **(E)** Sphere formation assay of mMC and mMPI cells in the 3D matrix for 5 days. Left, representative images of tumorsphere. Middle, relative surface area of tumorsphere. Right, quantification of sphere morphology. Scale bar, 100 μm. *n* = 3 biological replicates. Mean ± SD. * *P* < 0.05; ** *P* < 0.01; *** *P* < 0.001 by unpaired Student *t* test. **(F)** Quantification of lung metastatic loci in syngeneic mice 3 weeks after intravenous injection of mMC and mMPI cells (5 × 10^5^ cells per mouse). *n* = 6 mice per group. Mean ± SD. n.s., *P* > 0.05; * *P* < 0.05; ** *P* < 0.01 by unpaired Student *t* test. **(G)** Total PER and GlycoPER were measured in mMPI and mMC cells by glycolytic rate assay (GRA). Left, the curve of GRA. Middle, measurement of basal glycolysis, PER, and compensatory glycolysis. Right, the ratio of mitoOCR to glycoPER. Mean ± SD. n.s., *P* > 0.05; * *P* < 0.05; ** *P* < 0.01; *** *P* < 0.001 by unpaired Student *t* test. *n* = 3 biological replicates. Rot/AA, rotenone/antimycin A. 2-DG, 2-Deoxy-D-glucose. **(H)** Left, histogram showing the shift of MitoTracker Green signal in mMPI cells compared to mMC cells. Right, quantification of rela*t*ive MitoTracker intensity in mMC and mMPI cells. n.s., *P* > 0.05; * *P* < 0.05 by unpaired Student *t* test. *n* = 3 biological replicates. **(I–K)** Relative cell viability of mMC and mMPI cells under the different concentrations of 2-DG (I), Metformin (J), and UK-5099 (K). Mean ± SD. n.s., *P* > 0.05; * *P* < 0.05; ** *P* < 0.01; *** *P* < 0.001 by unpaired Student *t* test or nonlinear regression. *n* = 3 biological replicates. **(L)** Mito Fuel Flex was measured by Seahorse assay. **(M)** Mitochondria-derived ROS was measured with MitoSOX by flow cytometry in mMC and mMPI cells. Mean ± SD. n.s., *P* > 0.05; * *P* < 0.05; ** *P* < 0.01 by unpaired Student *t* test. *n* = 3 biological replicates. **(N)** Cellular general ROS level was measured in mouse melanoma cells (Left), A375 (Middle), and SK-MEL-28 (Right) cells by CellRox. Mean ± SD. n.s., *P* > 0.05; * *P* < 0.05; ** *P* < 0.01 by unpaired Student *t* tes*t*. *n* = 3 biological replicates. **(O)** Measurement of cellular NADPH/NADP^+^ ratio. Right, mMC vs. mMPI cells. Middle, mMC-EV vs. mMC-PLK1 cells. Left, A375 EV vs. A375 PLK1. Mean ± SD. n.s., *P* > 0.05; * *P* < 0.05; ** *P* < 0.01 by unpaired Student *t* test. *n* = 3 biological replicates. **(P)** Ratio of GSH/GSSG was measured. Left, mMC and mMPI cells upon the treatmen*t* of vehicle or 100 µM H_2_O_2_ for 24 h. Middle, A375 EV vs. A375 PLK1. Right, SK-MEL-28 EV vs. SK-MEL-28 PLK1. Mean ± SD. n.s., *P* > 0.05; * *P* < 0.05 by unpaired Student *t* test. *n* = 3 biological replicates. **(Q)** Relative cell viability of mMC and mMPI cells under the treatment of H_2_O_2_ after 72 h. Mean ± SD. n.s., *P* > 0.05; * *P* < 0.05; ** *P* < 0.01; *** *P* < 0.001 by unpaired Student *t* test. *n* = 3 biological replicates. The data underlying the graphs shown in the figure can be found in S1 Data.

To validate the function of PLK1 and understand its underlying mechanisms, we isolated and established 3 pairs of mouse melanoma stable cell lines from our GEM tumors. These cell lines were named mMC (for mouse melanoma cells) and mMPI (for mouse melanoma with Plk1 knock-in), respectively, derived from *Braf*^*CA/+*^ */ Pten*^*loxp/loxp*^ to *Braf*^*CA/+*^ */ Pten*^*loxp/loxp*^ */ Plk1* (Fig 2A). mMPI cells exhibited a marked increase in PLK1 expression compared to mMC cells, along with discernible differences in cell morphology and size (S2A and S2B Fig). mMPI cells showed an upregulation in migrative and invasive abilities, as well as the capacity to form colonies with low cell density, in comparison to mMC cells (Figs 2B and S2C). Similarly, the increased metastatic ability could be observed in the human melanoma cell line A375 and SK-MEL-28 with overexpressed PLK1 (Fig 2C and 2D). In addition, tumorspheres derived from mMPI cells grown in 3D Matrigel exhibited a significantly larger invasive area and more frequent stellate shape, indicating a faster growth rate and increased invasive capability compared to mMC cells [27] (Figs 2E and S2D). Meanwhile, the silencing of PLK1 in mouse melanoma cells attenuated the expression of the metastatic marker N-Cadherin (S2E Fig). To further validate the in vivo behavior of the PLK1-overexpressing cells, one of the representative mMC and mMPI cells was subjected to the mouse experiments. Consistent with our in vitro findings, increased PLK1 expression could enhance metastatic capability of mouse and human melanoma cells in vivo, leading to increased metastatic loci in lung tissues after intravenous inoculation (Figs 2F, S2F, and S2G).

### PLK1 triggers metabolic reprogramming in melanoma cells

Since melanoma metastasis is tightly correlated with metabolic adaptation [11,12], we evaluated the metabolic status in mMC and mMPI cells. According to the seahorse analysis, mMPI cells exhibited increasingly higher basal and compensatory glycolysis compared to mMC cells (Fig 2G). Intriguingly, even though there was no difference in basal mitochondrial respiration rate between mMC and mMPI cells, the mitoOCR/glycoPER ratio was reduced in mMPI cells, indicating that mMPI cells were more dependent on glycolysis for the energy supply (Figs 2G, S2H). In parallel, we observed the increased glycolytic capacity and reduced ATP production from respiration when overexpressed PLK1 in human melanoma cell A375 and its derived Vemurafenib-resistant A375R cells (S2I and S2J Fig). Since the RNA-seq results indicated the repression of OXPHOS in PLK1 overexpressed tumors, we measured the mitochondria mass and observed a significant decrease in mitochondrial mass in mMPI cells, revealing the suppression of mitochondrial biogenesis and OXPHOS in cells with high levels of PLK1 (Fig 2H). To further assess the metabolic profile of mMPI cells, we investigated their response to glycolysis inhibitor, 2-DG. Our findings indicated that mMPI cells exhibit a higher dependency on glucose and increased sensitivity to glycolytic inhibition (Fig 2I). In agreement, mMPI cells were significantly more resistant to metformin and UK-5099, inhibitors of OXPHOS, when compared to mMC cells (Fig 2J and 2K). Additionally, the Mito Fuel Flex assay supported that mMPI cells were more dependent on the glucose as the energy supply compared to the mMC cells (Fig 2L).

A recent publication has demonstrated the suppressive role of oxidative stress in the development of distant metastasis in melanoma [11]. Since OXPHOS is a major source of cellular ROS [28], we aimed to evaluate if higher PLK1 expression could impact the cellular ROS level. As expected, the frequency of cells with high mitochondria-derived ROS was about three times greater in mMC cells compared to mMPI cells, which implies high PLK1 expression suppress oxidative stress (Figs 2M, S2K). Besides, both mouse and human melanoma cells exhibited a similar pattern of decreased cellular general oxidative stress with overexpressed PLK1 (Fig 2N). NADPH and glutathione, two well-known antioxidant molecules, play a critical role in modulating redox homeostasis and promoting cell survival [29]. Our results showed that melanoma cells with higher PLK1 expression contributed to increased ratio of NADPH/NADP^+^ and GSH/GSSG in mouse and human melanoma cells, suggesting the induction of antioxidant ability (Fig 2O and 2P). Furthermore, mMPI cells exhibited greater detoxification ability towards extracellular ROS and increased tolerance to ROS inducers PMA and Etoxomir, with limited alteration in their intracellular ROS levels compared to mMC cells (Figs 2Q and S2L–S2P). In summary, our findings demonstrate that high PLK1 expression promotes metabolic reprogramming, reduces intracellular ROS levels, and enhances the antioxidant ability of melanoma cells.

### PLK1 contributes to Vemurafenib resistance in melanoma

**Fig 3:**
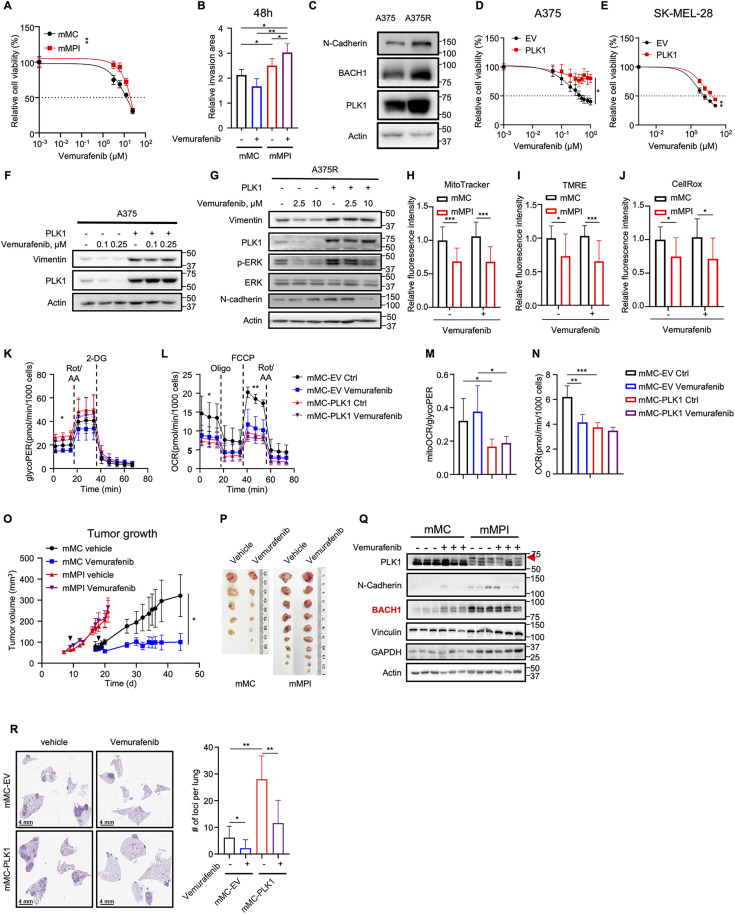
Polo-like kinase 1 (PLK1) induces Vemurafenib resistance in melanoma. **(A)** Relative cell viability under the treatment of Vemurafenib in mouse melanoma cells (mMC) and mouse melanoma with Plk1 knock-in (mMPI) cells. *n* = 3 biological replicates. Mean ± SD. n.s., *P* > 0.05; * *P* < 0.05; ** *P* < 0.01 by unpaired Student *t* test. **(B)** Quantification of spheroid invasion assay in mMC and mMPI cells under either vehicle or Vemurafenib treatment. *n* = 5 biological replicates. Mean ± SD. * *P* < 0.05; ** *P* < 0.01 by unpaired Student *t* test. **(C)** Immunoblot of PLK1 expression in human melanoma cell lines A375 and A375R. **(D and E)** Relative cell viability of human melanoma cells A375 (D) and SK-MEL-28 (E) under the treatment of different concentrations of Vemurafenib. *n* = 3 biological replicates. Mean ± SD. * *P* < 0.05; ** *P* < 0.01; *** *P* < 0. 001 by nonlinear regression. **(F and G)** Immunoblots of A375 (F) and A375R (G) cells under the treatment of Vemurafenib. **(H–J)** Mitochondria mass (H), mitochondria membrane potential (I), and cellular general ROS (J) were measured using MitoTracker Green, TMRE, and CellRox by flow cytometry in mMC and mMPI cells, respectively, upon the treatment of either vehicle or Vemurafenib. Mean ± SD. n.s., *P* > 0.05; * *P* < 0.05; ** *P* < 0.01; *** *P* < 0.001 by unpaired Student *t* test. *n* = 3 biological replicates. **(K)** Measurement of glycoPER in mMC-EV and mMC-PLK1 cells under the treatment of either vehicle or Vemurafenib by GRA. Mean ± SD. n.s., *P* > 0.05; * *P* < 0.05 by unpaired Student *t* test. *n* = 3 biological replicates. **(L)** Measurement of OCR in mMC-EV and mMC-PLK1 cells by Mito Stress test. Mean ± SD. n.s., *P* > 0.05; * *P* < 0.05; ** *P* < 0.01 by unpaired Student *t* test. *n* = 3 biological replica*t*es. Oligo, oligomycin. FCCP, carbonyl cyanide-4-(trifluoromethoxy)phenylhydrazone. **(M)** The ratio of mitoOCR/glycoPER was measured in the GRA in **(K)**. Mean ± SD. n.s., *P* > 0.05; * *P* < 0.05 by unpaired Student *t* test. *n* = 3 biological replicates. **(N)** ATP production was measured in the Mito Stress test in (L). Mean ± SD. n.s., *P* > 0.05; * *P* < 0.05; ** *P* < 0.01; *** *P* < 0.001 by unpaired Student *t* test. *n* = 3 biological replicates. **(O)** Tumor growth curve of subcutaneous implantation of mMC and mMPI cells to B6 mice (1 × 10^5^ cells per mouse), with the treatment of either vehicle or Vemurafenib (50 mg/kg body weight, oral gavage, once daily), respectively. *n* = 5, 7, 9, and 10, respectively. Mean ± SD. * *P* < 0.05 by unpaired Student *t* test. **(P)** Representative image of the freshly removed tumors at the end of the study. **(Q)** Immunoblot to analyze the indicated protein expression in the subcutaneous tumors of (O). Black arrow, PLK1 band. **(R)** Lung metastasis in syngeneic mice 3 weeks after intravenous injection (1 × 10^5^ cells per mouse). *n* = 7, 11, 8, and 12, respectively. Left, representative images of metastatic loci in the lung tissues. Right, quantification of tumor burden in lung tissues. Mean ± SD. * *P* < 0.05 by unpaired Student *t* test. Scale bar, 4 mm. The data underlying the graphs shown in the figure can be found in S1 Data.

Due to the clinical significance of Vemurafenib, sensitivity to Vemurafenib treatment was evaluated in mMC and mMPI cells. As indicated in Fig 3A, PLK1 overexpression contributed to drug resistance in mouse melanoma cells. Besides, the spheroid invasion assay confirmed the enhanced invasive ability and blunted drug efficiency in the mMPI cells (Figs 3B and S3A). In contrast to mMC cells, treatment of Vemurafenib even stimulated the invasion of tumor spheroids of mMPI cells in the 3D environment. The PLK1 protein level was also upregulated in the human Vemurafenib-resistant A375R cells, compared to its parental sensitive A375 cells, accompanied by increased expression of metastatic marker N-Cadherin (Fig 3C). Once PLK1 was overexpressed, human melanoma cells exhibited an attenuated response to Vemurafenib treatment (Figs 3D, 3E, and S3B), along with the increased expression of metastatic markers N-Cadherin and Vimentin (Fig 3F and 3G). In line with our previous finding, PLK1 overexpression impacts the metabolic status of melanoma, leading to the suppression of mitochondrial biogenesis and OXPHOS in cells (Figs 3H, 3I, S3C, and S3D). Previous studies have reported the contribution of ROS in the Vemurafenib-induced cell death [30]. In the presence of anti-ROS agent N-Acetyl Cysteine, the drug response was attenuated in mouse melanoma cells, which indicates that lower intracellular ROS level could contribute to the drug resistance (S3E Fig). Notably, cells with high levels of PLK1 exhibited much lower levels of intracellular ROS under the treatment of Vemurafenib, which may contribute, at least in part, to reduced drug response as discussed (Figs 3J, S3F, and S3G). Furthermore, overexpressing PLK1 in mMC cells contributed to the Warburg effect and induced the metabolic switch, with an increase in glycolysis but a suppression in OXPHOS (Figs 3K, 3L, S3H, and S3I). Remarkably, both mitoOCR/glycoPER ratio and mitochondria-involved ATP production reduced after overexpressing PLK1 in mMC cells (Fig 3M and 3N), revealing that PLK1 prompted the reliance on glycolysis. Under the stress of Vemurafenib, glycolysis and respiration were both significantly suppressed in mMC cells. In contrast, this effect was comparatively less pronounced in the PLK1 overexpressed cells. Additionally, the glycolytic inhibitor 2-DG sensitized mMPI cells to vemurafenib (S3J Fig), suggesting that enhanced glycolysis may partially contribute to PLK1-induced treatment resistance. On the contrary, the knockdown of *PLK1* by shRNA sensitized the cells to treatments and improved the efficacy of Vemurafenib in both sensitive parental cell A375 and resistant daughter A375R cells (S3K–S3M Fig). To further confirm PLK1’s role in vivo, we compared tumor proliferation and sensitivity to Vemurafenib treatment in a subcutaneous allograft (Figs 3O–3Q and S3N). In contrast to mMC cells, PLK1 overexpressed mMPI cells showed promoted tumor growth, significantly shortened doubling time, and limited response to Vemurafenib. Notably, mMPI-derived tumors also exhibited an elevated expression of metastatic markers N-Cadherin and Vinculin, as well as an upregulation of glycolytic protein GAPDH (Fig 3Q). Furthermore, in comparison to mMC-EV, mMC-PLK1 cells gained the metastatic potential to form more tumor burden in the lung tissues (Fig 3R). Compared to the mMC-EV cells, overexpressing PLK1 also led to the reduced efficacy of Vemurafenib. To conclude, these data indicate that PLK1 boosts the metastatic potential and diminishes the effectiveness of treatment in both human and mouse melanomas.

### Knockdown of BACH1 eliminates the PLK1-induced phenotypes in melanoma

**Fig 4:**
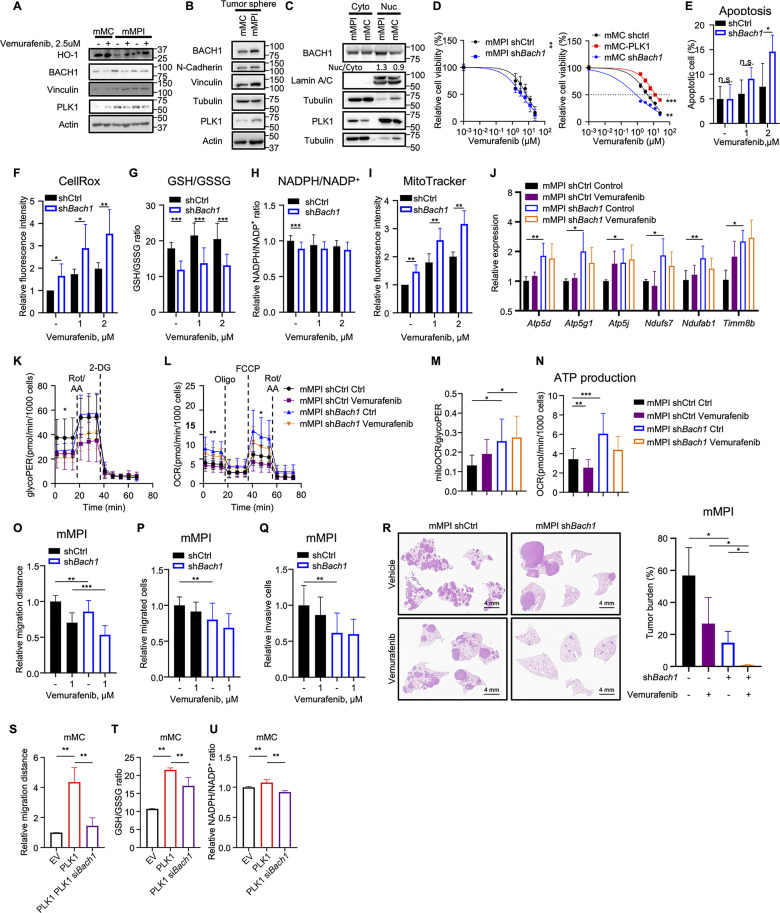
Knockdown of BTB domain and CNC homolog 1 (BACH1) impedes Polo-like kinase 1 (PLK1)-induced phenotype in melanoma. **(A and B)** Immunoblots for the expression of BACH1 in 2D-monolayer (A) and 3D-tumorsphere (B) of mouse melanoma cells (mMC) and mouse melanoma with Plk1 knock-in (mMPI) cells. **(C)** Immunoblots of BACH1 protein in nucleus and cytosol of mMC and mMPI cells. **(D)** Relative cell viability of mMPI (Left) and mMC (Right) under the treatment of Vemurafenib after BACH1 knockdown. Mean ± SD. n.s., *P* > 0.05; * *P* < 0.05; ** *P* < 0.01 by nonlinear regression. *n* = 3 biological replicates. **(E)** Percentage of apoptotic cells in mMPI shCtrl and mMPI sh*Bach1* cells after Vemurafenib treatment. Mean ± SD. n.s., *P* > 0.05; * *P* < 0.05 by unpaired Student *t* test. *n* = 3 biological replicates. **(F)** Cellular general ROS was assessed using the CellRox probe by flow cytometry in mMPI shCtrl and mMPI sh*Bach1* cells. Mean ± SD. n.s., *P* > 0.05; * *P* < 0.05; ** *P* < 0.01 by unpaired Student *t* test. *n* = 3 biological replicates. **(G and H)** GSH/GSSG (G) and NADPH/NADP^+^ (H) ratios were analyzed in mMPI shCtrl and mMPI sh*Bach1* cells. Mean ± SD. n.s., *P* > 0.05; * *P* < 0.05; ** *P* < 0.01; *** *P* < 0.001 by unpaired Student *t* test. *n* = 3 biological replicates. **(I)** The mitochondrial mass of mMPI shCtrl and mMPI sh*Bach1* cells was analyzed by MitoTracker Green. Mean ± SD. n.s., *P* > 0.05; * *P* < 0.05; ** *P* < 0.01; *** *P* < 0.001 by unpaired Student *t* test. *n* = 3 biological replicates. (**J**) qRT-PCR analysis was performed to detect the mRNA level of BACH1 downstream targets in mMPI shCtrl and mMPI sh*Bach1* cells. Mean ± SD. n.s., *P* > 0.05; * *P* < 0.05; ** *P* < 0.01 by unpaired Student *t* test. *n* = 3 biological replicates. **(K)** GlycoPER was measured by GRA in mMPI shCtrl and mMPI sh*Bach1* cells. Mean ± SD. n.s., *P* > 0.05; * *P* < 0.05 by unpaired Student *t* test. *n* = 3 biological replicates. **(L)** Measurement of OCR by Mito Stress test in mMPI shCtrl and mMPI sh*Bach1* cells. Mean ± SD. n.s., *P* > 0.05; * *P* < 0.05; ** *P* < 0.01 by unpaired Student *t* test. *n* = 3 biological replicates. (**M**) mitoOCR/glycoPER was calculated by GRA in (K). Mean ± SD. n.s., *P* > 0.05; * *P* < 0.05 by unpaired Student *t* test. *n* = 3 biological replicates. **(N)** ATP production was calculated by the Mito Stress test in (L). Mean ± SD. n.s., *P* > 0.05; * *P* < 0.05; ** *P* < 0.01; *** *P* < 0.001 by unpaired Student *t* test. *n* = 3 biological replicates. **(O–Q)** The metastatic potential of mMPI shCtrl and mMPI sh*Bach1* cells were analyzed by wound healing assay (O), transwell migration assay (P), and transwell invasion assay (Q). Mean ± SD. n.s., *P* > 0.05; * *P* < 0.05; ** *P* < 0.01; *** *P* < 0.001 by unpaired Student *t* test. *n* = 3 biological replicates. (**R**) mMPI shCtrl and mMPI sh*Bach1* cells were intravenously injected into the syngeneic mice (1 × 10^5^ cells per mouse). After 1 week, the mice were treated with either vehicle or Vemurafenib by oral gavage (50 mg/kg body weight, once daily), respectively. The mice were sacrificed for tissue collection af*t*er 4 weeks. *n* = 5, 5, 10, and 11, respectively. Left, representative images of metastatic loci in the lung tissues. Right, quantification of tumor burden in lung tissues. Mean ± SD. * *P* < 0.05 by unpaired Student *t* test. Scale bar, 4 mm. **(S)** Wound healing assay was performed in mMC-PLK1 cells after silencing *Bach1*. Mean ± SD. n.s., *P* > 0.05; * *P* < 0.05; ** *P* < 0.01; *** *P* < 0.001 by unpaired Student *t* test. *n* = 3 biological replicates. **(T and U)** GSH/GSSG (T) and NADPH/NADP^+^ (U) were analyzed after knockdown BACH1 in PLK1-overexpressed mMC cells. Mean ± SD. n.s., *P* > 0.05; * *P* < 0.05; ** *P* < 0.01; *** *P* < 0.001 by unpaired Student *t* test. *n* = 3 biological replicates. The data underlying the graphs shown in the figure can be found in S1 Data.

To identify the key molecule associated with PLK1 in melanoma, we initially examined transcription factors that are closely linked to OXPHOS. Interestingly, our search led us to BACH1, which not only regulates the electron transport chain (ETC) but also directly upregulates the transcription of genes involved in glycolysis and metastasis [31,32]. BACH1 is a member of the CNC-bZip family and can compete with NRF2 for binding to antioxidant response elements in response to changes in redox status [33,34]. Recent research has revealed that BACH1 plays a crucial role in shaping the metabolic and metastatic potential of cancer cells during cancer progression, irrespective of its antagonism with NRF2 [35]. Next, we analyzed the top-regulated genes from the RNA-seq results using Gene Cards and found that the promoter or enhancer regions of most of these genes had a binding element for BACH1 (S1 Table) [36,37], suggesting BACH1 as a potential mediator of the phenomenon that we observed. We subsequently examined the protein expression of BACH1 in both mouse melanoma cells and tumors. Consistent with the results from GEM and subcutaneous tumors (Figs 3Q and S1I), we observed a significant increase in BACH1 expression in mMPI cells, both in adherent 2D monolayer and 3D tumorspheres (Fig 4A and 4B). Notably, unlike mMC cells, BACH1 predominantly accumulated in the nucleus of mMPI cells rather than in the cytosol, indicating a potential enhanced transcriptional activity of BACH1 in these cells (Fig 4C).

To further dissect the role of BACH1 in PLK1-associated phenotypes, we established stable cell lines with BACH1 knockdown by shRNA. The efficacy of shRNA was validated by qRT-PCR toward *Bach1* and its classic downstream target *Hmox1*, which suggested the successful repression of BACH1 in mMPI cells (S4A and S4B Fig). Additionally, immunoblot analysis revealed a decreased BACH1 protein level, with no obvious impact on PLK1 expression (S4C Fig). Reducing BACH1 expression in both mMPI and mMC cells resulted in increased sensitivity to Vemurafenib, leading to decreased cell viability and enhanced apoptosis (Fig 4D and 4E). Moreover, when BACH1 was abolished, there was a significant increase in cellular ROS levels observed in both human and mouse melanoma cells, coupled with a reduction in antioxidant molecules NADPH and GSH (Figs 4F–4H and S4D–S4F). Furthermore, mMPI cells expressing sh*Bach1* showed an increase in mitochondria mass and ETC-related genes, concomitant with a decrease in glycolysis and an increased dependency on OXPHOS (Figs 4I–4N and S4G). Upon Vemurafenib treatment, BACH1-depleted mMPI cells showed increased cellular ROS levels, reduced antioxidants, elevated mitochondria mass, and enhanced OXPHOS, which triggered excessive oxidative stress accompanied by enhanced proliferative suppression and massive cell apoptosis. Moreover, the reduction of *Cdh2* expression in mMPI cells was attributed to BACH1 knockdown (S4H Fig), indicating the inhibition of metastasis and invasion with lower BACH1. This finding was further corroborated by wound healing and transwell assays on the mouse and human melanoma cells in vitro (Figs 4O–4Q and S4I–S4N). Subsequently, the metastatic potential was assessed by metastatic loci formed in lung tissues by mMPI shCtrl and mMPI sh*Bach1* cells following the tail vein injection in syngeneic mice (Fig 4R). Consistently, the reduced expression of BACH1 eliminated the cells’ metastatic ability and enhanced the efficacy of Vemurafenib in vivo. Furthermore, BACH1 was silenced by siRNA to identify if the PLK1-induced phenotype could be rescued in the mMC-PLK1 cells. As expected, the knockdown of BACH1 in mMC-PLK1 cells reduced the metastatic potential and antioxidant ability (Figs 4S–4U and S4O). Taken together, our results demonstrate that PLK1 promotes melanoma development and progression through BACH1, while the depletion of BACH1 could abolish the PLK1-induced phenotypes in melanoma.

### PLK1 contributes to the stabilization of BACH1

**Fig 5:**
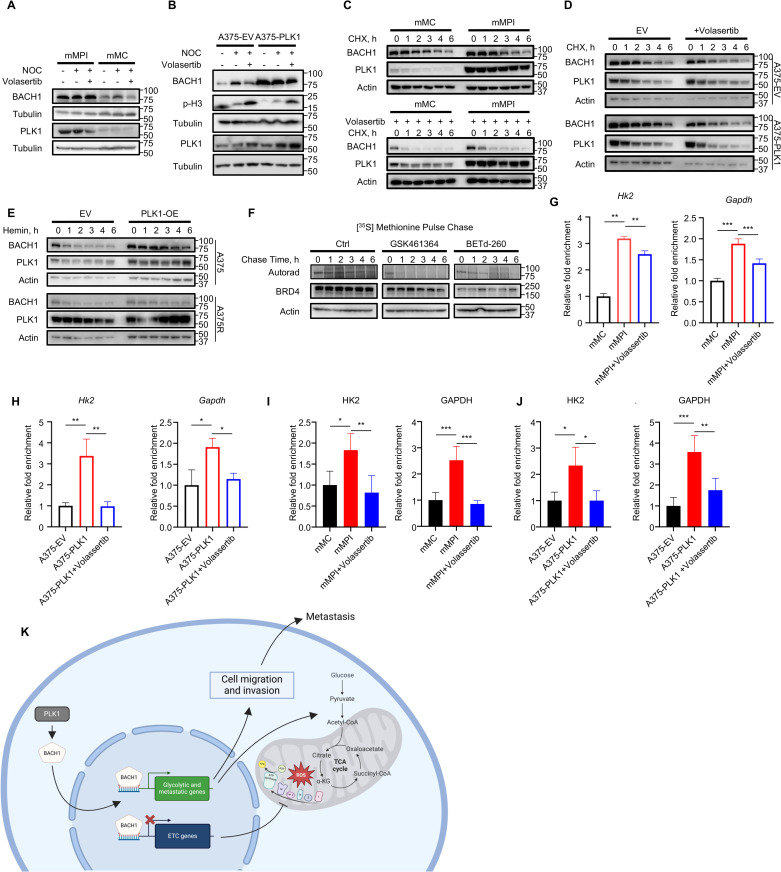
BTB domain and CNC homolog 1 (BACH1) is stabilized by Polo-like kinase 1 (PLK1). **(A and B)** BACH1 protein level was detected in mouse (A) and human (B) melanoma cells under the treatment of either DMSO, Nocodazole (NOC), or NOC plus Volasertib. **(C and D)** Immunoblots of BACH1 protein level in the mouse (C) and human (D) melanoma cells under the treatment of Cycloheximide (CHX) at indicated time points with or without Volasertib. **(E)** Human melanoma cells were treated with Hemin and collected at the indicated time points, followed by the immunoblots to determine the BACH1 protein. **(F)** [^35^S]-methionine pulse chase assay was performed in the A375R cells. (**G and H**) mRNA expression level of GAPDH and HK2 was analyzed by qRT-PCR in mouse (G) and human (H) melanoma cells. Mean ± SD. n.s., *P* > 0.05; * *P* < 0.05; ** *P* < 0.01 by unpaired Student *t* test. *n* = 3 biological replicates. **(I and J)** Chromatin-immunoprecipitation (ChIP) was performed to detect the chromatin occupancy of BACH1 in mouse (I) and human (J) melanoma cells. Mean ± SD. n.s., *P* > 0.05; * *P* < 0.05; ** *P* < 0.01 by unpaired Student *t* test. *n* = 3 biological replicates. **(K)** A working model to illustrate how PLK1 promotes melanoma progression through BACH1. PLK1 could prevent BACH1 from proteasome degradation, resulting in hyper-activation of BACH1. Consequently, the downstream targets of BACH1 were regulated accordingly, thus promoting metabolic reprogramming and metastasis in melanoma. This figure was created with BioRender.com. The data underlying the graphs shown in the figure can be found in S1 Data.

To investigate the regulatory role of PLK1 on BACH1 in melanoma, we assessed the protein stability of BACH1 and found its protein level significantly reduced in melanoma cells under the PLK1 inhibition (S5A Fig). To eliminate the potential effect of the cell cycle when PLK1 is most abundant, we arrested cells at the G2/M phase using nocodazole (NOC) and investigated the expression of BACH1 in the presence or absence of PLK1. Compared to the NOC-only group, Volasertib, a PLK1 inhibitor, could lead to a decreased BACH1 protein level (Figs 5A, 5B, and S5B). To further assess the impact of PLK1 on BACH1, CHX (Cycloheximide) chase assay was performed to check the stability of BACH1 protein. In mouse and human melanoma cells with PLK1 overexpression, BACH1 demonstrated a significantly more stable phenotype and an extended half-life (Figs 5C, 5D, S5C, and S5D). However, treatment with the PLK1 inhibitor Volasertib accelerated BACH1 degradation, rendering it unstable even in the context of high PLK1 expression. Similarly, upon Hemin treatment, an inducer of BACH1 degradation [21], PLK1 overexpression led to more stabilized BACH1 in the melanoma cells (Figs 5E and S5E). Volasertib has been reported to exhibit bromodomain activity, which may interfere with the BRD4 activity as a global protein transcriptional regulator [38]. To rule out this potential off-target effect, BACH1 protein stability was further assessed using an [^35^S]-methionine pulse chase assay. Compared to the untreated control group, treatment with another PLK1 inhibitor, GSK461364, which lacks bromodomain cross-reactivity [38], could still accelerate BACH1 degradation, resulting in a shorter protein half-life. In contrast, the BRD4 degrader BETd-260 had minimal effect on BACH1 protein levels, as shown in Fig 5F.

Since BACH1 is a transcription factor to regulates multiple gene expression, we investigated whether reduced BACH1 protein level could influence its transcriptional activity. We first examined the mRNA levels of BACH1 downstream targets GAPDH and HK2 (Fig 5G and 5H). With overexpressed PLK1, the expression of these targets was significantly boosted in melanoma cells, the effect of which was abolished by the addition of PLK1 inhibitor. In addition, the chromatin occupancy of BACH1 at the promoter regions of GAPDH and HK2 was further assessed by ChIP assay (Fig 5I and 5J). Aligned with the nuclear accumulation of BACH1 observed in Fig 4C, high-level expression of PLK1 enhanced the transcriptional activity of BACH1 in the promoter region, and its effect could be reversed by PLK1 inhibition. Combined with these observations, we conclude that PLK1 promotes the metabolic switch, metastasis, and Vemurafenib resistance in melanoma through regulation of BACH1, as illustrated in Fig 5K.

### Inhibition of PLK1 significantly improves the efficacy of Vemurafenib in melanoma

**Fig 6:**
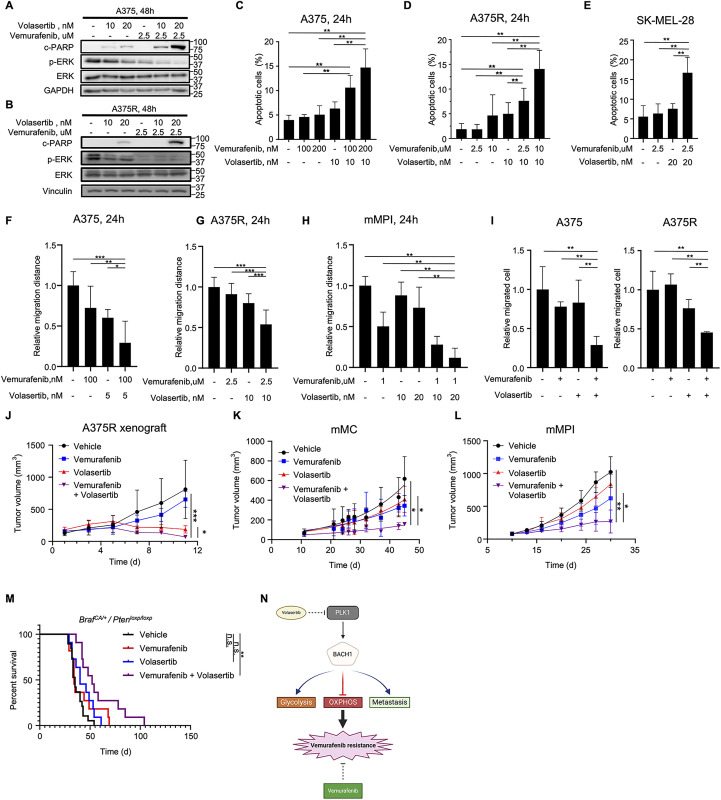
Inhibition of Polo-like kinase 1 (PLK1) synergizes with Vemurafenib in the treatment of melanoma. **(A and B)** Immunoblots of cleaved-PARP (c-PARP) and p-ERK detected in A375 (A) and A375R (B) cells after 48 h treatment of either DMSO, Vemurafenib, Volasertib, or a combination of Vemurafenib and Volasertib, respectively. **(C–E)** Percentages of apoptotic cells were measured by Annexin V staining after 48 h treatment of indicated drugs in human melanoma cells A375 (C), A375R (D), and SK-MEL-28 (E). Mean ± SD. n.s., *P* > 0.05; * *P* < 0.05; ** *P* < 0.01 by unpaired Student *t* test. *n* = 3 biological replicates. **(F–H)** Metastatic ability was assessed by wound healing assay in A375 (F), A375R (G), and mMPI (H) cells, upon the 24 h treatment of indicated drugs. Mean ± SD. n.s., *P* > 0.05; * *P* < 0.05; ** *P* < 0.01; *** *P* < 0.001 by unpaired Student *t* test. *n* = 3 biological replicates. **(I)** Transwell migration assay of A375 and A375R cells under drug treatmen*t*. **(J)** Growth curves of A375R-derived tumors. A375R cells (2 × 10^6^) were subcutaneously inoculated into the right flank region of female nude mice, followed by the administration with either vehicle, Vemurafenib (50 mg/kg body weight, oral gavage, once daily), Volasertib (10 mg/kg body weight, intraperitoneal injection, every 2 days), or a combination of both drugs. Mean ± SD. n.s., *P* > 0.05; * *P* < 0.05; ** *P* < 0.01; *** *P* < 0.001 by unpaired Student *t* test. *n* = 4 mice from each experimental group. **(K and L)** Growth curves of mMC (K) and mMPI (L)-derived tumors. Female B6 mice were subcutaneously inoculated with mouse melanoma cells (1 × 10^6^) and administrated with the vehicle, Vemurafenib, Volasertib, or the combination of Vemurafenib and Volasertib after 1 week. Mean ± SD. n.s., *P* > 0.05; * *P* < 0.05; ** *P* < 0.01; *** *P* < 0.001 by unpaired Student *t* test. *n* = 3 and 10, respectively, for each experimental group of mMC and mMPI. **(M)** Long-term survival analysis of *Braf*^*CA/+*^ */ Pten*^*loxp/loxp*^ GEM mice upon the treatment of indicated drugs. *n* = 19, 11, 11, and 11, respectively, for the vehicle, Vemurafenib, Volasertib, or the dual treatment. The mice were euthanized when the tumor maximum diameter reached 14 mm. n.s., *P* > 0.05; * *P* < 0.05; ** *P* < 0.01 by Log-rank (Mantel–Cox) test. **(N)** A proposed working model to reveal the mechanism of synergy between Vemurafenib and Volasertib. The figure was generated with BioRender.com. The data underlying the graphs shown in the figure can be found in S1 Data.

According to our prior observations that the PLK1/BACH1 axis contributes to the Vemurafenib resistance in melanoma, we investigated whether the PLK1 inhibitor Volasertib, in combination with Vemurafenib, could achieve improved therapeutic efficacy in melanoma. Compared to the treatment with Vemurafenib alone, combination with Volasertib dramatically suppressed the MAPK signaling pathway with a reduction of p-ERK signal but also induced massive cell apoptosis in human melanoma cells (Fig 6A–6E). Besides, Volasertib and Vemurafenib co-treatment significantly rendered colony formation ability and cell proliferation rate in A375 and A375R cells, in comparison to the monotherapies (S6A–S6D Fig). We next calculated the synergy between Volasertib and Vemurafenib by the combination index (CI) using the Chou–Talalay method [39], which was 0.667 and 0.75, respectively, in A375 and A375R cells (S2 and S3 Tables), demonstrating the strong synergetic effect between these two drugs. In parallel, the synergy between Vemurafenib and Volasertib was abolished in the A375R cells with BACH1 knockout via CRISPR (S6E Fig and S4 Table). With minimal expression of BACH1, the combination treatment exhibited an additive effect, yielding a CI score of 0.977, which highlights the critical role of BACH1 in mediating this synergy. To investigate if the co-treatment could affect the cell metastasis, the metastatic ability was evaluated by in vitro wound healing assay and transwell migration assays (Figs 6F–6I, S6F, and S6G). While Vemurafenib alone barely impacted cell migration, the combination with Volasertib enhanced the effectiveness of Vemurafenib and further diminished the metastatic potential of melanoma cells in vitro.

To further confirm the aforementioned in vitro findings, we next validated the synergy and efficacy of Volasertib and Vemurafenib in vivo using several mouse models. In the A375R-derived xenograft experiment, due to the Vemurafenib resistance, tumors presented a very limited response toward the single treatment of Vemurafenib compared to the vehicle group (Figs 6J and S6H). Although Volasertib alone can suppress tumor proliferation, combining it with Vemurafenib further enhances its efficacy in inhibiting tumor proliferation. In addition, combination therapy deeply represses several key regulators of melanoma development compared to either the vehicle or single-treatment groups. These regulators include MAPK signaling pathway indicators p-BRAF and p-ERK, metastatic markers N-Cadherin and Vimentin, proliferative marker PCNA, and melanoma-specific transcription factor SOX10 (S6I Fig). Histological analysis demonstrated a significant decrease in cellularity and a marked increase in apoptotic bodies in the dual-treated groups (S6J Fig). Additionally, tumors from the combined treatment group also exhibited a reduction in cellular proliferation, as demonstrated by the proliferation marker Ki-67, and showed an increase in apoptosis, as demonstrated by the apoptotic marker cleaved-Caspase 3 (S6J Fig). Consistently, we observed a similar suppressive trend of tumor proliferation with a prolonged tumor doubling time upon the co-treatment of Volasertib and Vemurafenib in the mMC and mMPI-derived allograft (Figs 6K, 6L, S6K, and S6L). Notably, the long-term survival analysis revealed that the dual inhibition of PLK1 and BRAF^V600E^ successfully prolonged the survival period in *Braf*^*CA/+*^ */ Pten*^*loxp/loxp*^ mice (Fig 6M). Although neither Volasertib nor Vemurafenib alone could achieve this effect, the combination of both treatments resulted in significant survival benefits, suggesting that this novel therapeutic strategy could have potential translational applications in the clinic.

## Discussion

PLK1, a serine/threonine kinase involved in cell cycle regulation, is known to participate in various cancer-associated signaling pathways [14]. Accumulating evidence has revealed that PLK1 is highly elevated in multiple cancer types and correlated with worse patient survival [23]. More specifically, previous studies have demonstrated the contribution of overexpressed PLK1 to cancer development in mouse models of lymphoma and lung cancer [24,40]. To identify the role of PLK1 in melanoma, we analyzed patient data from the TCGA database. Our analysis has uncovered that high *Plk1* is associated with a shortened survival period in patients with melanoma, suggesting a tumor-promoting role of PLK1 in melanoma progression. Next, we evaluated the role of PLK1 in the *Braf*^*CA/+*^ */ Pten*^*loxp/loxp*^ mouse melanoma model and modulated the *Plk1* expression by crossing it with either *Plk1-*KI or *Plk1*^*loxp/loxp*^ mice [21,22]. In comparison to *Braf*^*CA/+*^ */ Pten*^*loxp/loxp*^ mice, overexpression of *Plk1* resulted in accelerated melanoma progression with a high proliferative rate, higher frequency of metastasis, and worse survival. In contrast, the depletion of *Plk1* significantly improved overall survival. Importantly, overexpression of PLK1 promoted the Warburg effect, metastasis, and Vemurafenib resistance in human and mouse melanoma cells. Our results suggest that these PLK1-associated phenotypes were mediated through BACH1, a master transcription factor for metastasis and metabolism, which was stabilized by PLK1. Therefore, these findings support the notion of an oncogenic role of *Plk1* in the development and progression of melanoma.

Metastatic melanoma is a highly lethal cancer that is accompanied by reversible metabolic changes to adapt to the altered microenvironment and provide the necessary energy supply [2]. In recent studies, it has been shown that elevated mitochondrial activity, OXPHOS, and increased reactive oxidative stress can suppress melanoma metastasis [10–12]. Thus, antioxidants such as NADPH and GSH play a crucial role in protecting against oxidative stress and are necessary for cancer initiation and progression [11,41,42]. Vemurafenib, an FDA-approved inhibitor targeting BRAFV600E, has significantly improved the survival of patients with metastatic melanoma, but its effectiveness is limited in terms of duration [6]. Interestingly, a study suggested that reduced mitochondrial biogenesis may contribute to intrinsic resistance to MAPKi in a subgroup of melanoma cells with BRAF mutation [43]. This highlights the crucial role of mitochondria and OXPHOS in treatment resistance. In addition to its classical role in the cell cycle, multiple publications revealed the crosstalk between PLK1 and MAPK signaling, implying their potential mutual promotion of activity [16,40]. In addition, PLK1 has also been linked to metabolic regulation, suggesting that PLK1 is significantly associated with glycolysis and the pentose phosphate pathway [15,44,45]. However, the role of PLK1 in melanoma metastasis and metabolism is elusive and requires further investigation. Of note, RNA-seq results of our study have revealed that overexpression of *Plk1* promotes metastasis and metabolic reprogramming in melanoma GEM tumors. Consistent with the prior publications, *Plk1* overexpressing tumors exhibited high metastatic potential accompanied by significant suppression of OXPHOS and response to oxidative stress. Additionally, mMPI cells, derived from *Braf*^*CA/+*^ */ Pten*^*loxp/loxp*^ */ Plk1* mice, showed a markedly increased metastatic capability and Vemurafenib resistance, compared to *Braf*^*CA/+*^ */ Pten*^*loxp/loxp*^–derived mMC cells. Similarly, higher *Plk1* expression contributed to metabolic reprogramming with elevated dependency on glycolysis rather than OXPHOS in vitro. Moreover, oxidative stress was repressed in mMPI cells compared to mMC cells, suggesting a potential mechanism for PLK1-induced drug resistance and metastasis. Although PLK1 and MAPK signaling are correlated, the link between OXPHOS and MAPK signaling is indirectly and not fully established. Our research aims to uncover a novel mechanism that elucidates how PLK1 influences metabolism based on our findings.

BACH1 is a critical transcription factor that contains a bZip domain and shares the same DNA binding domain with NRF2 in response to oxidative response [33,34]. Previously, BACH1 was known as a transcriptional repressor due to its ability to recruit the corepressors NCOR1, NCOR2, and the histone deacetylases to its DNA binding regions [46,47]. Notably, BACH1 directly binds to heme via cysteine-proline motifs in the intrinsically disordered region, leading to the nuclear export and subsequent proteasomal degradation [48]. Recent studies have demonstrated that BACH1 promotes metastasis and EMT in multiple types of cancers [31,32,49]. In addition, BACH1 participates in the regulation of metabolism via suppressing OXPHOS while stimulating glycolysis [20,21], thus leading to the Warburg effect in the cancer cells. The activity of BACH1 could potentially be regulated by posttranslational modifications, such as phosphorylation by kinases [50,51]. Mass spectrometry has identified multiple phosphorylation sites of BACH1, the majority of which still have unknown functions and require further investigation [51]. In the present study, we observed BACH1 was highly expressed in PLK1 overexpressed cells and tumor samples. Importantly, the knockdown of BACH1 abolished the phenotypes elicited by PLK1 in both human and melanoma cells, with reduced glycolysis, increased OXPHOS, inhibited metastasis, and resensitization to Vemurafenib treatment. In summary, our study has revealed that BACH1 could be regulated by PLK1, thus preventing its degradation.

Vemurafenib is a front-line drug used to treat metastatic melanoma in patients, particularly those with BRAF^V600E^ mutations, as it has demonstrated a high response rate and improved survival compared to chemotherapy [6,52]. Despite the initial benefits of Vemurafenib, the challenge of extending its duration of effectiveness and enhancing its efficacy for patients remains. Clinical trials have investigated various combination therapies to improve the effectiveness of BRAF inhibitors in the treatment of metastatic melanoma, such as the combination of MEK inhibitors and immune checkpoint blockade [52–54]. Although these combination therapies have shown promising results, resistance to treatment can still emerge after a period of time [53]. Herein, we reported a novel PLK1/BACH1 axis as a resistant mechanism in the treatment of melanoma with Vemurafenib. Based on our working model (Fig 6N), inhibition of PLK1 leads to decreased expression of BACH1, which activates OXPHOS, produces excessive oxidative stress, and inhibits metastasis, resulting in increased sensitivity to Vemurafenib. We validated the efficacy of PLK1 inhibitor Volasertib in combination with Vemurafenib both in vitro and in vivo, demonstrating strong synergy in suppressing human and mouse melanoma. Our study not only highlights PLK1 as a biomarker for poor prognosis in malignant melanoma but also establishes it as a valuable therapeutic target for enhancing the efficacy of Vemurafenib in clinical settings.

## Materials and methods

### Mouse models

*Braf*^*CA/+*^ */ Pten*^*loxp/loxp*^*/ Tyr::CreER*^*T2*^ mice were purchased from the Jackson lab. *Plk1* mice were generated by our lab as previously described [24]. *Plk1*^*loxp/loxp*^ mice were kindly provided by Dr. Guillermo de Cárcer from Spanish National Cancer Research Centre (CNIO), Madrid, Spain [25]. Both female and male mice were used in the experiments. For the localized tumor induction, 4-OHT (Sigma, Cat No. 579002) was dissolved in 100 mM stock solution by DMSO. Upon tumor induction, the 4-OHT stock solution was further diluted by ethanol to 20 mM, and 1–2 µL of the solution was applied to the skin of the mouse back region [21,22].

### Cell culture

A375 and A375R cells were kind gifts by Dr. Nihal Ahmad from the University of Wisconsin, USA. SK-MEL-28 cell was kindly provided by Dr. John A. D’Orazio from the University of Kentucky, USA. Mouse melanoma cell lines mMC and mMPI were isolated from the GEM tumors. HEK293T and B16-F10 cells were purchased from ATCC. All the cell lines were cultured in DMEM with 10% fetal bovine serum at 37 °C in 5% CO_2_. All the cells were within 50 passages, and Mycoplasma was detected every 3 months using the MycoAlert PLUS Mycoplasma Detection Kit (Lonza, Cat No. LT07-705).

For 3D culture, DMEM/F12 medium was used and supplemented with 20 ng/mL EGF, 20 ng/mL bFGF, 10% B27, 4 µg/mL insulin, and 0.4% bovine serum albumin. For the tumorsphere assay, the 96-well plate was pretreated with Matrigel (Corning, 354230) and incubated at 37 °C for 1 h. The cells were suspended using FBS-free medium, mixed with Matrigel at 1:1 to 100 µL in total, and then seeded into the pre-coated 96-well plates. Finally, 50 µL DMEM/F12 medium was added in the top, and the cells were cultured at 37 °C in 5% CO_2_. The tumorsphere was monitored, and photos were taken on day 3 and day 5. For the spheroid invasion assay, the cells were suspended in DMEM/F12 medium and generated spheroid using the hanging drop method. After 4 days of incubation, the spheroids were transferred to the 96-well plate with 50 μL medium, with or without drugs, and then 50 μL Matrigel was added to each well. The plate was incubated for 1 h for the Matrigel to solidify, and 100 μL of the medium was added [55].

### Antibodies and reagents

Antibodies against SOX10 (sc-365692), p-ERK (sc7383), ERK (sc-514302), HO-1(sc-390991), TFAM (sc-166965), NOX1 (sc-51802), SOD2 (sc-137254), and Catalase (sc-271803) were products of Santa Cruz Biotechnology. Anti-PLK1 (05-884) and p-H3 (06-570) were obtained from Millipore Sigma. Antibodies to Tubulin (T5168), FLAG (F1804), and Vinculin (V4505) were purchased from Sigma. Anti-BACH1 (14018-1-AP) was obtained from Proteintech. Antibodies against GPX4 (ab125066) and Ki-67 (ab16667) were purchased from Abcam. Antibodies to GAPDH (2118), N-Cadherin (13116), Vimentin (5741), Actin (4970), HA (3724), BRAF (14814), p-BRAF (2696), PCNA (2586), cleaved-PARP (5625), and cleaved-Caspase 3 (9661) were purchased from Cell Signaling Technology.

Vemurafenib (HY-12057), Volasertib (HY-12137), PMA (HY-18739), Metformin (HY-B0627), Etomoxir (HY-50202), UK-5099 (HY-15475), Hemin (HY-19424), GSK461364 (HY-50877), and BETd-260 (HY-101519) were obtained from MedChem Express. Cycloheximide (S7418) and Nocodazole (S2775) were purchased from Selleckchem.

The human BACH1 cDNA clone (RG209275) was from OriGene. shRNAs targeting mouse *Bach1* (TRCN0000288208, TRCN0000295567, TRCN0000295568), Human *BACH1* (TRCN0000013593, TRCN0000013596, TRCN0000013597), and mouse *Plk1* (TRCN0000274592, TRCN0000274637) were purchased from Sigma. shRNAs targeting human *PLK1* were constructed and described previously [56]. siRNAs targeting mouse *Bach1* were also purchased from Sigma. sgRNAs targeting human *BACH1* were purchased from ABM (Cat. No. 12974111). The transfection reagent jetPRIME (101,000,046) was purchased from Polyplus.

### Cell viability assay

Cells (2 × 10^3^–1 × 10^4^ per well) were seeded into 96-well plates, cultured overnight, and then treated with the indicated drugs in a series of different concentrations. After 72 h of incubation, AquaBluer (MultiTarget, 6015) was added to the medium and incubated for 4 h. Later, the absorbance was measured at 570 nm by a plate reader. The IC_50_ values were obtained from the average viability curves generated by at least three independent measurements under each condition.

### Combination index

The CI was calculated by using the following equation: $\text{CI}\;=\;\frac{(\text{Am}textrm50}{(\text{As})\text{50}}+\frac{(\text{Bm})\text{50}}{(\text{Bs})\text{50}}$ with the Chou–Talalay method [39]. The IC_50_ of each drug was measured, respectively, as (As)_50_ and (Bs)_50_. Subsequently, the two drugs were mixed to treat the cells, to get the parameters of (Am)_50_ and (Bm)_50_ when it achieved a 50% inhibitory effect. CI > 1 indicates antagonism, CI = 1 indicates addition, and CI < 1 indicates synergy.

### Transwell migration and invasion assay

For the migration assay, cells were suspended using the serum-free medium and seeded into a transwell insert for 2 × 10^4^–4 × 10^4^ cells per well. For the invasion assay, the Matrigel (Corning, 354230) was diluted with the serum-free medium in the ratio of 1:40 to pretreat the transwell inserts for 1 h under 37 °C. After seeding cells, a medium containing 10% FBS was added to the receiver well. The cells were incubated for 24 h and then fixed with 10% formalin. The inserts were then stained with 0.5% crystal violet for 30 min and washed with PBS several times [57]. Finally, the photos of the transwell were taken using a Nikon microscope.

### Wound healing assay

Cells were seeded into 6-well plates and incubated overnight. Once the cell density achieved 100% confluence, a vertical wound was made by a 200 μL pipette tip down through the cell monolayer. The cells were washed three times with PBS and then cultured in the medium containing 1% serum or 50 ng/mL recombinant EGF (PeproTech, 315-09) with or without drugs. The pictures were taken at 12 h or 24 h with the Nikon microscope to monitor the wound closure [57].

### Colony formation assay

Cells (500–5000/well) were cultured in the medium alone or containing different drugs for 14 days, and the medium was changed every 2 days. Upon harvest, colonies were fixed in 10% formalin and stained with 0.5% crystal violet for 30 min. The colonies were washed with PBS several times, and the colony numbers were counted.

### Flow cytometry analysis

Cells were seeded, cultured overnight, and treated with either DMSO or the indicated drugs for 24 h or 48 h. For Annexin V staining, the cells were trypsinized, collected, washed with PBS, and then subjected to the procedure using the Annexin V apoptosis kit (Biolegend, 640926). For MitoSOX (ThermoFisher, M36008), MitoTracker Green FM (Cell Signaling Technology, 9074), TMRE (Cayman Chemical, 701310), CellRox (ThermoFisher, C10422), and CM-H_2_DCFDA (ThermoFisher, C400) staining, the probes were added into the medium and incubated for 15–30 min according to the manufacturer’s manual. Cells were then collected, stained with DAPI (ThermoFisher, D1306), and subjected to flow cytometry analysis.

### Immunoblotting

Cells were washed with PBS twice and lysate with RIPA buffer containing inhibitors of protease and phosphatase. After sonication and centrifugation, cell lysates were collected, and protein concentrations were measured by the Pierce BCA Protein Assay Kit (ThermoFisher, 23225). After quantification, all samples were adjusted to equal total protein amounts, mixed with SDS–PAGE loading buffer, and denatured at 95 °C for 5 min. The protein samples were loaded and run in the SDS–PAGE gel. After transferring to polyvinylidene difluoride membranes, proteins of interest were probed with corresponding primary antibodies diluted in 5% milk in a 1:1000 ratio. After overnight incubation at 4 °C, the membrane was washed 3 times for 5 min with TBST and incubated with diluted second antibodies (1:3000) at room temperature for 2 h. Before exposure, the membrane was washed 3 times for 5 min with TBST, and then the signal was detected with the Clarity Western ECL Substrate (BIO-RAD, Cat No. 1705061). The loading control was probed and detected in the same membrane as the target protein.

### [^35^S]-methionine pulse chase assay

A375R cells were pretreated with either DMSO, GSK461364, or BETd-260, then starved in methionine-free medium for 1 h, followed by pulse labeling with [^35^S]-methionine (100 μCi/mL) for an additional hour. After labeling, cells were washed and cultured in complete medium containing CHX, in the presence of either DMSO, GSK461364, or BETd-260. Cells were harvested at the indicated time points and subjected to immunoprecipitation or western blot analysis.

### RNA isolation, quantitative real-time PCR (qRT-PCR), and RNA sequencing (RNA-seq)

Total RNA was extracted from either tissues or cells by using the RNeasy mini kit (Qiagen, 74104) according to the manufacturer’s manual. For RNA-seq, the RNA was extracted from different mice tumors. *n* = 6 and 5 biological replicates, respectively, for *Braf*^*CA/+*^ */ Pten*^*loxp/loxp*^ and *Braf*^*CA/+*^ */ Pten*^*loxp/loxp*^ */ Plk1* mice. The extracted RNA was then submitted to Novegene Biotechnology Company (CA, USA) for quality control assessment, library construction, Illumina sequencing, and data analysis. Readcount was obtained from Gene Expression Analysis and then used for differential expression analysis. DESeq2 R package was used to perform the Gene expression data normalization and differential expression analysis. Fold change ≥ 2 and *q*-value < 0.05 were recognized as significantly regulated genes. GSEA was then performed using GSEA software version 4.1.0 (Broad Institute) with rank-ordered gene lists. For qRT-PCR, 1 μg RNA was subjected to reverse transcription using the QuantiTect Reverse Transcription Kit (Qiagen, 205314) following the kit manual. FastStart Universal SYBR Green Master was used to measure the expression level of indicated mRNA and was normalized to *Actb*. The primers for qRT-PCR are listed in S5 Table. To analyze potentially correlated transcription factors, significantly differentially expressed genes (DEGs) were first identified from the DEG list with a fold change ≥ 2 and an adjusted *P*-value (padj) < 0.01. Mouse-specific genes were excluded, and the remaining gene list was submitted for query on the GeneCards website to retrieve information on related transcription factors.

### ChIP-qPCR

Human and mouse melanoma cells were fixed with 37% formaldehyde for 10 min and quenched with 0.125M glycine. Chromatin was isolated with the SimpleChIP Plus Enzymatic Chromatin IP Kit (Magnetic Beads) (9005, CST), and was immunoprecipitated with BCAH1 antibody (14018-1-AP, ProteinTech), or IgG (2729, CST). Samples were subjected to qPCR analysis with the specific primers listed in S6 Table.

### Hematoxylin and eosin (H&E) staining and Immunohistochemistry (IHC) staining

Tumors were fixed in 10% formalin, embedded in paraffin, and sectioned at 5 μm by the Biospecimen Procurement and Translational Pathology Shared Resource Facility (BPTP SRF) at the University of Kentucky. H&E staining was also performed by the BPTP SRF. For IHC staining, after deparaffinization and rehydration of paraffin-embedded slides, the antigens of tissues were retrieved in antigen unmasking solution (Vector Laboratories, H-3301-250). Samples were then blocked, and incubated with indicated primary antibodies in a 1:200 ratio, followed by incubation with secondary antibodies. Next, the slides were stained with VECTASTAIN Elite ABC Universal Plus kit (Vector Laboratories, PK-8200) and Harris’s hematoxylin (Vector Laboratories H-3401-500). All the pictures were taken with a Nikon microscope. The image scanning was performed by the BPTP SRF using the Aperio Digital Pathology Slide Scanners.

### Human cell xenograft and mouse cell allograft

For subcutaneous implantation, the human or mouse melanoma cells (1 × 10^5^–2 × 10^6^ cells per mouse) were mixed with Matrigel (Corning, 356232), and injected subcutaneously into the right flank of either female NSG or B6 mice, respectively. Once the tumor volume achieved 100 mm^3^, the mice were randomized to each group, followed by the treatment of either vehicle or the indicated drugs. Tumor volumes were estimated from the formula: V = L × W^2^/2 [V is the volume (mm^3^); L is the length (mm); W is the width (mm)]. For intravenous injection, the human or mouse melanoma cells (1 × 10^5^–1 × 10^6^ cells per mouse) were resuspended in PBS and intravenously injected into the tail vein of NSG or B6 mice, respectively. The mice were randomly separated and treated with the vehicle or the indicated drug after one week. The weight was monitored, and the mice were sacrificed once they met the criteria for euthanization. The experiment was approved by University of Kentucky Animal Care and Use Committee (IACUC). Approved protocol No. is 2020-3681.

### Lentiviral production

To produce lentiviruses, HEK293T cells were transfected with lentiviral backbone constructs and packaging vectors using JetPrime transfection reagent. For human lentivirus, psPAX2 and pMD2.G were used for viral packaging. For mouse lentivirus, delta8.2 and VSVG were used as the packaging vectors. psPAX2 (Addgene plasmid # 12260), pMD2.G (Addgene plasmid # 12259), and pCMV delta R8.2 were gifts from Didier Trono (Addgene plasmid # 12263). pCMV-VSV-G was a gift from Bob Weinberg (Addgene plasmid # 8454). Lentiviruses were collected 48 hours post-transfection and centrifuged. Afterward, the supernatant was collected and passed through 0.45 μm membrane filters. The lentiviruses were then added to the melanoma cells. After 48 h of infection, the cells were resuspended and cultured in the medium containing selective antibiotics.

### Seahorse analysis

Melanoma cells (5 × 10^3^−1.5 × 10^4^) were seeded in the Seahorse XF96 V3 PS Cell Culture Microplates (Agilent Technologies, 101085-004) and treated with DMSO or the indicated drug overnight. Next, the plates were submitted to the Redox Metabolism Facility at the University of Kentucky for Seahorse XF Glycolytic Rate Assay, Mito Stress Test, and Mito Fuel Flex assay, using the Seahorse XFe96 Analyzer. The cell number for each well was counted by Hoechst staining. All the seahorse data were normalized with cell numbers.

### TCGA database analysis of melanoma patients

Data analysis was performed by GEPIA [58] and the Biostatistics and Bioinformatics Shared Resource Facility at the University of Kentucky. Melanoma patient data were obtained from TCGA (https://cancergenome.nih.gov/). For normal skin samples, the data were obtained from the GTEx portal (https://gtexportal.org/home/). In the comparison of tumor versus normal samples, all gene expressions have been converted to Transcripts per Million expressions to account for variability in the library size of samples. All statistical analyses and corresponding figures were conducted in R-4.0.0. The Kaplan–Meier method and the log-rank test were used to compare survival time and tumor progression between high and low gene expression subgroups, with the median expression of PLK1 as the threshold.

### NADPH/NADP

The cells were seeded into 24-well plates and cultured overnight. Subsequently, they were treated with either DMSO or the indicated drugs for 48 h. Upon cell harvest, the Promega NADP/NADPH-Glo Assay kit (G9081) was used, and the measurement was performed following the manufacturer’s manual.

### GSH/GSSG assay

The cells were seeded in 96-well plates and cultured overnight, followed by treatment with either DMSO or the indicated drugs for 48 h. GSH/GSSG levels were measured using the Promega GSH/GSSG-Glo Assay kit (V6611) according to the manufacturer’s instructions.

### Nuclear extract assay

The melanoma cells were seeded to 100 mm dishes and cultured overnight. Once it achieved 100% confluency, the cells were collected and washed twice with cold PBS. Next, the nuclear and cytosol protein was extracted using the Nuclear Extract Kit (Active Motif, 40010). All the procedures were performed according to the manufacturer’s instructions.

### Statistical analysis

All results and graphs were statistically analyzed and generated by GraphPad Prism 8 software (GraphPad Software USA). Numerical data were analyzed using the unpaired Student *t* test. The statistical information is described in the corresponding figure legends. *P* < 0.05 indicates statistical significance. Numerical data are presented as Mean ± SD. Reproducibility was ensured by performing at least three independent experiments.

## Supporting information

S1 Fig RNA-seq results reveal PLK1’s role in melanoma development. (PDF)

S2 Fig PLK1 induces melanoma development in vitro and in vivo. (PDF)

S3 Fig Overexpression of PLK1 impacts the treatment response. (PDF)

S4 Fig BACH1 depletion reverses PLK1-associated phenotype in melanoma. (PDF)

S5 Fig BACH1 could be stabilized by PLK1. (PDF)

S6 Fig PLK1 inhibitor enhances the efficacy of Vemurafenib in vitro and in vivo. (PDF)

S1 Table List of transcription factors associated with the top-regulated genes. (XLSX)

S2 Table The IC_50_ values of Volasertib and Vemurafenib in A375 cells. (DOCX)

S3 Table The IC_50_ values of Volasertib and Vemurafenib in A375R cells. (DOCX)

S4 Table The IC_50_ values of Volasertib and Vemurafenib in A375R-sgBACH1 cells. (DOCX)

S5 Table Primers for qRT-PCR. (DOCX)

S6 Table Primers for ChIP-qRT. (DOCX)

S1 Raw image Raw images of Figs 1–6 and S1–S6 Figs. (PDF)

S1 Data Numerical values of data in Figs 1–6 and S1–S6 Figs. (XLSX)

## Abbreviations

4-OHT: 4-Hydroxy-Tamoxifen
BACH1: BTB domain and CNC homolog 1
BPTP SRF: Biospecimen Procurement and Translational Pathology Shared Resource Facility
CHX: Cycloheximide
CI: combination index
DEG: differentially expressed gene
EMT: epithelial-mesenchymal transition
ETC: electron transport chain
GEM: genetically engineered mouse
GRA: Glycolytic Rate Assay
GSEA: gene set enrichment analysis
H&E: hematoxylin and eosin
IHC: Immunohistochemistry
MAPK: mitogen-activated protein kinase
mMC: mouse melanoma cells
mMPI: mouse melanoma with Plk1 knock-in
NOC: nocodazole
OXPHOS: oxidative phosphorylation
PLK1: Polo-like kinase 1
qRT-PCR: quantitative real-time PCR
RNA-seq: RNA sequencing
ROS: reactive oxygen species
TCGA: The Cancer Genome Atlas
