# Supplementary material for: The cell cycle regulator PLK1 promotes murine melanoma progression by regulating the transcription factor BACH1
Source: PLoS Biol. 2025 Nov 24;23(11):e3003490. doi: 10.1371/journal.pbio.3003490 (PMC12643297; doi:10.1371/journal.pbio.3003490)
Supplement: S1 Fig — (PDF) [file pbio.3003490.s001.pdf]

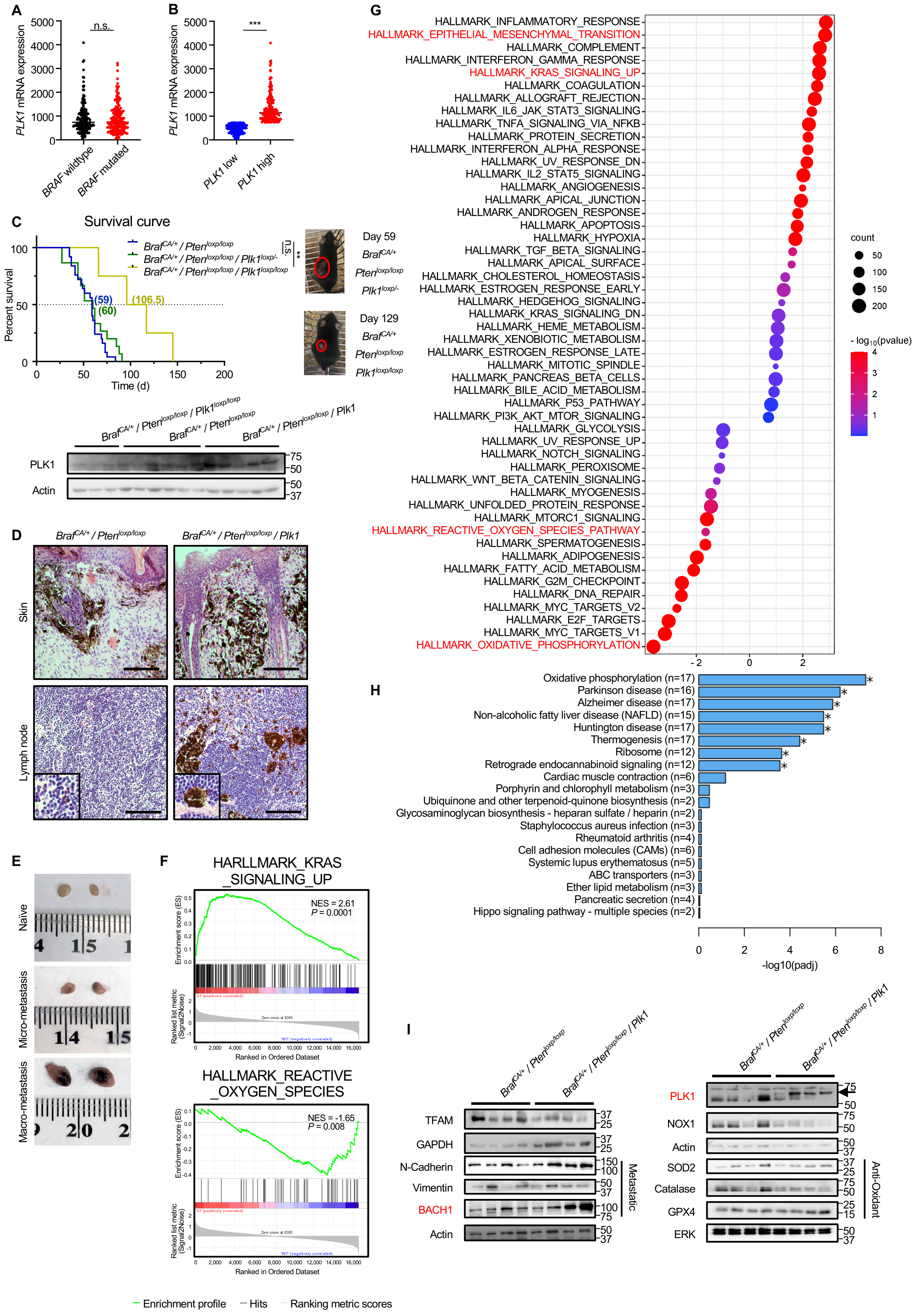
A
PLK1 mRNA expression
n.s.
BRAF wildtype
BRAF mutated
B
***
PLK1 low
PLK1 high
C
Survival curve
Percent survival
Time (d)
(59)
(60)
(106.5)
Day 59
Day 129
PLK1
Actin
D
Skin
Lymph node
E
Naïve
Micro-metastasis
Macro-metastasis
F
HARLLMARK_KRAS _SIGNALING_UP
NES = 2.61
P = 0.0001
HALLMARK_REACTIVE _OXYGEN_SPECIES
NES = -1.65
P = 0.008
Enrichment score (ES)
Ranked list metric (Signal2Noise)
Ranked in Ordered Dataset
Enrichment profile
Hits
Ranking metric scores
G
HALLMARK_INFLAMMATORY_RESPONSE
HALLMARK_EPITHELIAL_MESENCHYMAL_TRANSITION
HALLMARK_COMPLEMENT
HALLMARK_INTERFERON_GAMMA_RESPONSE
HALLMARK_KRAS_SIGNALING_UP
HALLMARK_COAGULATION
HALLMARK_ALLOGRAFT_REJECTION
HALLMARK_IL6_JAK_STAT3_SIGNALING
HALLMARK_TNFA_SIGNALING_VIA_NFKB
HALLMARK_PROTEIN_SECRETION
HALLMARK_INTERFERON_ALPHA_RESPONSE
HALLMARK_UV_RESPONSE_DN
HALLMARK_IL2_STAT5_SIGNALING
HALLMARK_ANGIOGENESIS
HALLMARK_APICAL_JUNCTION
HALLMARK_ANDROGEN_RESPONSE
HALLMARK_APOPTOSIS
HALLMARK_HYPOXIA
HALLMARK_TGF_BETA_SIGNALING
HALLMARK_APICAL_SURFACE
HALLMARK_CHOLESTEROL_HOMEOSTASIS
HALLMARK_ESTROGEN_RESPONSE_EARLY
HALLMARK_HEDGEHOG_SIGNALING
HALLMARK_KRAS_SIGNALING_DN
HALLMARK_HEME_METABOLISM
HALLMARK_XENOBIOTIC_METABOLISM
HALLMARK_ESTROGEN_RESPONSE_LATE
HALLMARK_MITOTIC_SPINDLE
HALLMARK_PANCREAS_BETA_CELLS
HALLMARK_BILE_ACID_METABOLISM
HALLMARK_P53_PATHWAY
HALLMARK_PI3K_AKT_MTOR_SIGNALING
HALLMARK_GLYCOLYSIS
HALLMARK_UV_RESPONSE_UP
HALLMARK_NOTCH_SIGNALING
HALLMARK_PEROXISOME
HALLMARK_WNT_BETA_CATENIN_SIGNALING
HALLMARK_MYOGENESIS
HALLMARK_UNFOLDED_PROTEIN_RESPONSE
HALLMARK_MTORC1_SIGNALING
HALLMARK_REACTIVE_OXYGEN_SPECIES_PATHWAY
HALLMARK_SPERMATOGENESIS
HALLMARK_ADIPOGENESIS
HALLMARK_FATTY_ACID_METABOLISM
HALLMARK_G2M_CHECKPOINT
HALLMARK_DNA_REPAIR
HALLMARK_MYC_TARGETS_V2
HALLMARK_E2F_TARGETS
HALLMARK_MYC_TARGETS_V1
HALLMARK_OXIDATIVE_PHOSPHORYLATION
count
- log10(pvalue)
H
Oxidative phosphorylation (n=17)
Parkinson disease (n=16)
Alzheimer disease (n=17)
Non-alcoholic fatty liver disease (NAFLD) (n=15)
Huntington disease (n=17)
Thermogenesis (n=17)
Ribosome (n=12)
Retrograde endocannabinoid signaling (n=12)
Cardiac muscle contraction (n=6)
Porphyrin and chlorophyll metabolism (n=3)
Ubiquinone and other terpenoid-quinone biosynthesis (n=2)
Glycosaminoglycan biosynthesis - heparan sulfate / heparin (n=2)
Staphylococcus aureus infection (n=3)
Rheumatoid arthritis (n=4)
Cell adhesion molecules (CAMs) (n=6)
Systemic lupus erythematosus (n=5)
ABC transporters (n=3)
Ether lipid metabolism (n=3)
Pancreatic secretion (n=4)
Hippo signaling pathway - multiple species (n=2)
-log10(padj)
I
TFAM
GAPDH
N-Cadherin
Vimentin
BACH1
Actin
Metastatic
PLK1
NOX1
Actin
SOD2
Catalase
GPX4
ERK
Anti-Oxidant

S1 Fig. RNA-Seq results reveal PLK1's role in melanoma development.

(**A**) *PLK1* mRNA level in the melanoma patients harboring either wildtype *BRAF* or mutated *BRAF*. *P* value by unpaired student's *t* test. (**B**) *PLK1* expression level in the melanoma patients with either low or high *PLK1* expression. *P* value by unpaired student's *t* test. (**C**) Survival curve of $Braf^{CA/+}$ / $Pten^{loxp/loxp}$ mice versus $Braf^{CA/+}$ / $Pten^{loxp/loxp}$/ $Plk1^{loxp/-}$ mice versus $Braf^{CA/+}$ / $Pten^{loxp/loxp}$ / $Plk1^{loxp/loxp}$ mice, *n* = 25, 15, and 4, respectively. The mice were euthanized when the average diameter of tumors reached 15 mm. n.s., $P > 0.05$; *, $P < 0.05$; **, $P < 0.01$ by Log-rank (Mantel-Cox) test. Right, representative images of tumors formed in $Braf^{CA/+}$ / $Pten^{loxp/loxp}$ / $Plk1^{loxp/-}$ mouse and $Braf^{CA/+}$ / $Pten^{loxp/loxp}$ / $Plk1^{loxp/loxp}$ mouse after 59d and 129d induction, respectively. Bottom, western blot showing the Plk1 expression level in melanoma tumors from indicated genotypes. (**D**) H&E staining of localized induced mouse tumors. Top, skin tissues. Bottom, lymph nodes. Scale bar, 150 μm. (**E**) Representative images of mouse lymph nodes with different extents of melanoma metastasis. (**F**) GESA analysis of KRAS_SIGNALING_UP (Top) and REACTIVE_OXYGEN_SPECIES (Bottom) pathways with alternatively expressed genes in tumors of $Braf^{CA/+}$ / $Pten^{loxp/loxp}$ / *Plk1* versus $Braf^{CA/+}$ / $Pten^{loxp/loxp}$. Normalized enrichment scores (NES) and nominal *P* values are shown. (**G**) Bubble plot based on the GSEA analysis of hallmark gene sets. (**H**) Bar chart of KEGG pathway enrichment. (**I**) Immunoblotting for the expression of indicated proteins in the melanoma tumors from $Braf^{CA/+}$ / $Pten^{loxp/loxp}$ / *Plk1* and $Braf^{CA/+}$ / $Pten^{loxp/loxp}$ mice. Black arrow, PLK1. The data underlying the graphs shown in the figure can be found in S1 Data.
