# Supplementary material for: The cell cycle regulator PLK1 promotes murine melanoma progression by regulating the transcription factor BACH1
Source: PLoS Biol. 2025 Nov 24;23(11):e3003490. doi: 10.1371/journal.pbio.3003490 (PMC12643297; doi:10.1371/journal.pbio.3003490)
Supplement: S2 Fig — (PDF) [file pbio.3003490.s002.pdf]

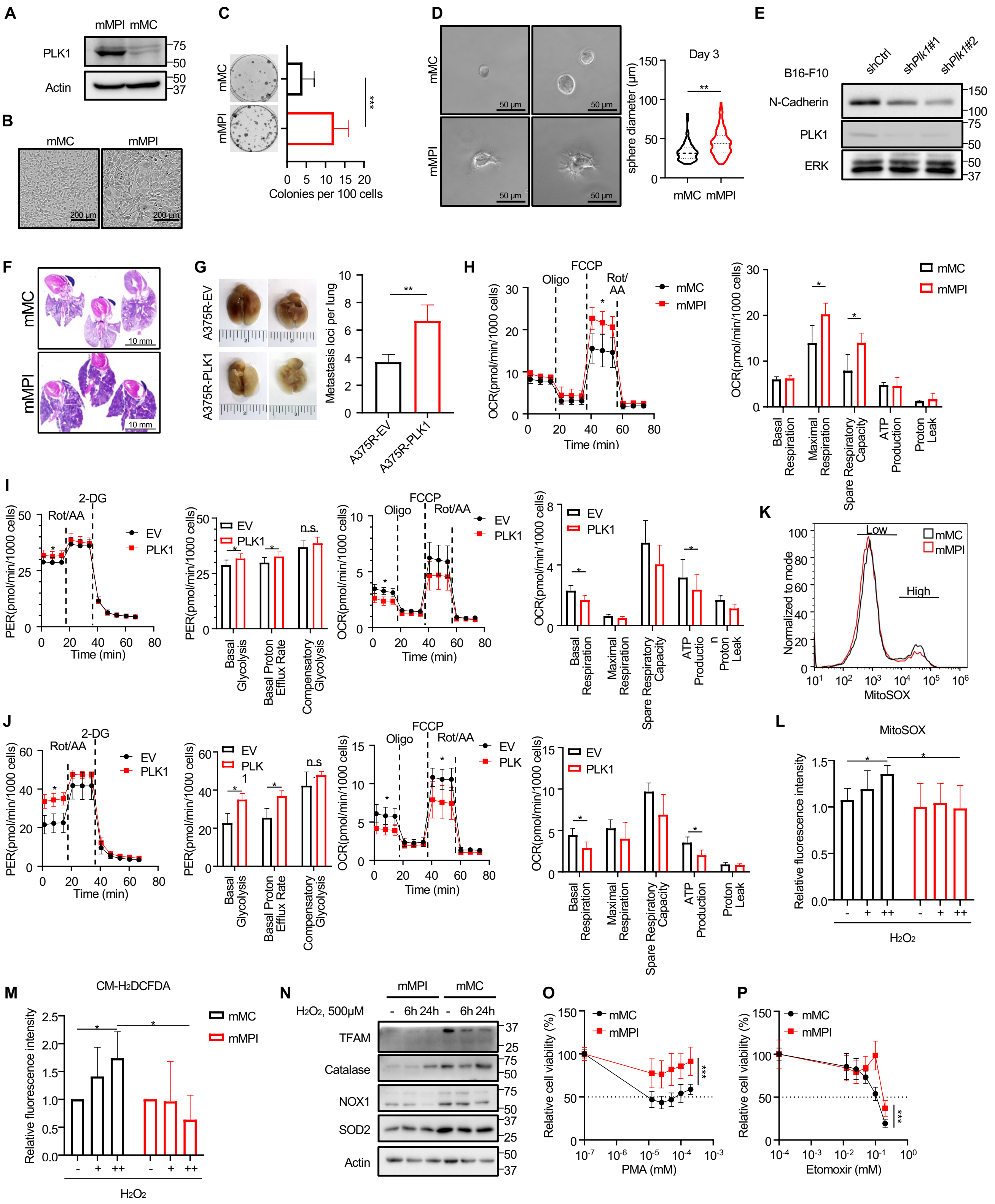
A
mMPI mMC
PLK1
Actin
B
mMC
mMPI
C
mMC
mMPI
Colonies per 100 cells
D
mMC
mMPI
Day 3
sphere diameter (μm)
mMC mMPI
E
B16-F10
shCtrl
shPlk1#1
shPlk1#2
N-Cadherin
PLK1
ERK
F
mMC
mMPI
G
A375R-EV
A375R-PLK1
Metastasis loci per lung
H
Oligo
FCCP
Rot/AA
mMC
mMPI
OCR(pmol/min/1000 cells)
Time (min)
Basal Respiration
Maximal Respiration
Spare Respiratory Capacity
ATP Production
Proton Leak
I
Rot/AA
2-DG
EV
PLK1
PER(pmol/min/1000 cells)
Basal Glycolysis
Basal Proton Efflux Rate
Compensatory Glycolysis
K
Low
High
Normalized to mode
MitoSOX
J
L
MitoSOX
Relative fluorescence intensity
H2O2
M
CM-H2DCFDA
N
H2O2, 500μM
- 6h 24h - 6h 24h
TFAM
Catalase
NOX1
SOD2
O
Relative cell viability (%)
PMA (mM)
P
Etomoxir (mM)

S2 Fig. PLK1 induces melanoma development in vitro and in vivo

(**A**) Immunoblots of PLK1 expression in mMC and mMPI cells. (**B**) Representative images of mMC and mMPI cells. Scale bar, 200 µm. (**C**) Colony formation of mMC and mMPI cells. Left, representative images of colonies. Right, quantification of colonies formed per 100 cells. n = 6 biological replicates. Mean ± SD. *, $P < 0.05$; **, $P < 0.01$; ***, $P < 0.001$ by unpaired student's *t*. (**D**) Sphere formation assay of mMC and mMPI cells in the 3D matrix for 3 days. Left, representative images of tumor sphere. Right, quantification of tumor sphere diameter. Scale bar, 50 µm. n = 3 biological replicates. Mean ± SD. *, $P < 0.05$; **, $P < 0.01$ by unpaired student's *t*. (**E**) Immunoblots for expression of N-Cadherin in mouse melanoma cell line B16-F10 upon knockdown of PLK1 by shRNA. (**F**) H&E staining of lung metastasis in syngeneic mice 3 weeks after intravenous injection of mMC and mMPI cells (5×10^5 cells per mouse). n = 6 mice per group. Scale bar, 10 mm. (**G**) Lung metastasis in NSG mice 3 weeks after intravenous injection of either A375R-EV or A375R-PLK1 cells (5×10^5 cells per mouse). n = 3 mice per group. Left, representative images of lung tissues freshly removed from mice. Right, quantification of metastatic loci found in each lung. Mean ± SD. n.s., $P > 0.05$; *, $P < 0.05$; **, $P < 0.01$ by unpaired student's *t* test. (**H**) Measurement of OCR in mMC and mMPI cells by Mito Stress test. Left, OCR curve during the Mito Stress test. Right, Oxidative phosphorylation parameters measured in Mito Stress test. Mean ± SD. n.s., $P > 0.05$; *, $P < 0.05$ by unpaired student's *t* test. n = 3 biological replicates. (**I** and **J**) GRA and Mito Stress were tested in A375 (**I**) and A375R (**J**) respectively. Mean ± SD. n.s., $P > 0.05$; *, $P < 0.05$ by unpaired student's *t* test. n = 3 biological replicates. (**K**) Histogram of MitoSox staining in mMC and mMPI cells. Based on the intensity, the whole cell population could be separated into low and high groups as illustrated in the graph. (**L** and **M**) Mitochondria-derived ROS and cellular general ROS levels were detected by MitoSox (**L**) and CM-H_2DCFDA (**M**) in mMC and mMPI cells upon 24h treatment of H_2O_2. Mean ± SD. n.s., $P > 0.05$; *, $P < 0.05$ by unpaired student's *t*. n = 3 biological replicates. (**N**) Immunoblots of mMC and mMPI cells under treatment of H_2O_2. (**O** and **P**) Relative cell viability of mMC and mMPI cells under the 72h treatment of PMA (**O**) and Etomoxir (**P**). Mean ± SD. n.s., $P > 0.05$; *, $P < 0.05$; **, $P < 0.01$; ***, $P < 0.001$ by unpaired student's *t* test. The data underlying the graphs shown in the figure can be found in S1 Data.
