# Supplementary material for: The cell cycle regulator PLK1 promotes murine melanoma progression by regulating the transcription factor BACH1
Source: PLoS Biol. 2025 Nov 24;23(11):e3003490. doi: 10.1371/journal.pbio.3003490 (PMC12643297; doi:10.1371/journal.pbio.3003490)
Supplement: S3 Fig — (PDF) [file pbio.3003490.s003.pdf]

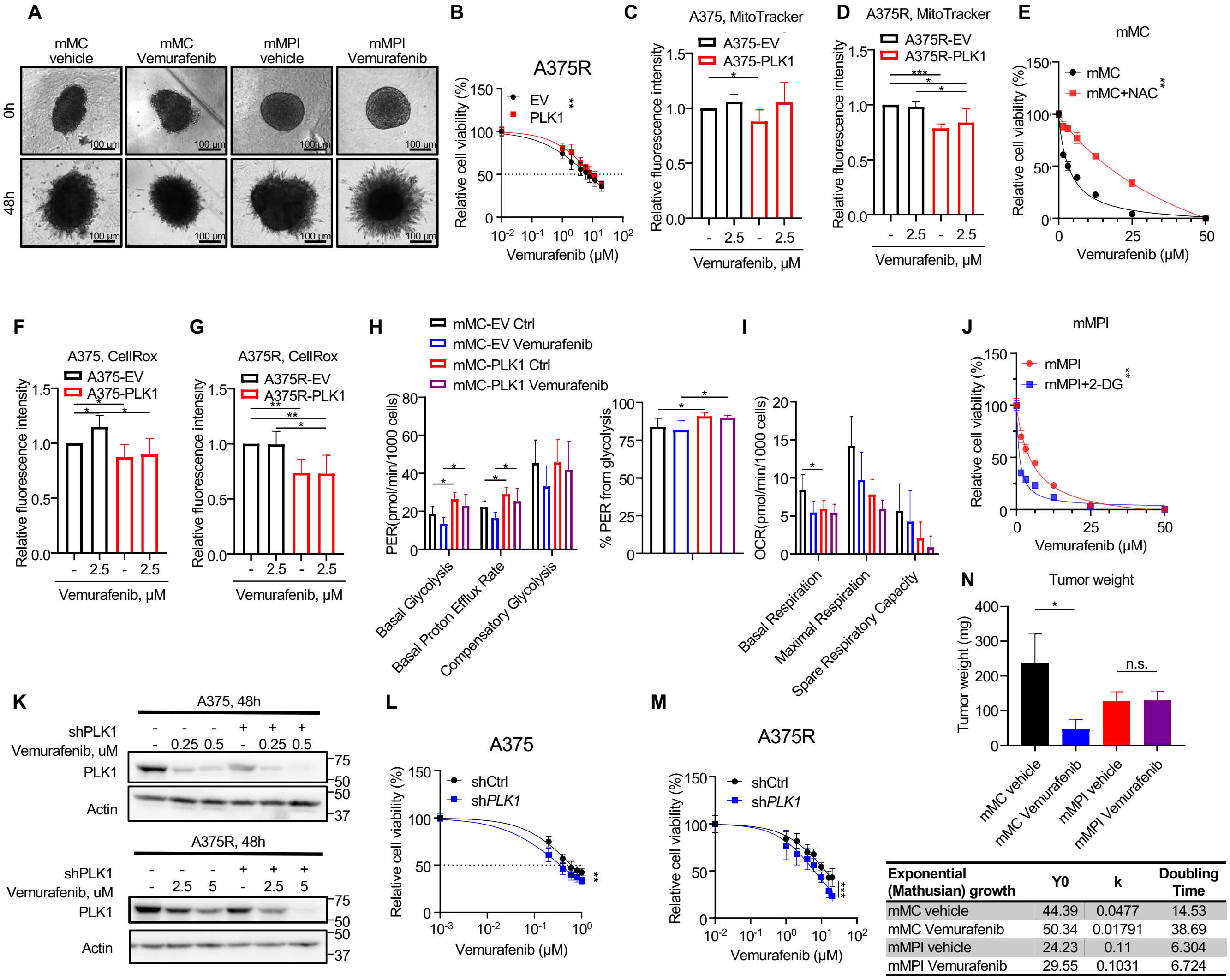

Exponential (Mathusian) growth	Y0	k	Doubling Time
mMC vehicle	44.39	0.0477	14.53
mMC Vemurafenib	50.34	0.01791	38.69
mMPI vehicle	24.23	0.11	6.304
mMPI Vemurafenib	29.55	0.1031	6.724

S3 Fig. Overexpression of PLK1 impacts the treatment response

(A)Representative images of spheroid invasion assay. Scale bar, 100 μm. (**B**) Relative cell viability of A375R-EV and A375R-PLK1 under the treatment of Vemurafenib for 72h. Mean ± SD. n.s., $P > 0.05$; *, $P < 0.05$; **, $P < 0.01$ by non-linear regression. (**C** and **D**) Mitochondria mass was measured using MitoTracker Green by flow cytometry after 24h treatment of either vehicle or Vemurafenib. (**C**) A375-EV versus A375-PLK1. (**D**) A375R-EV versus A375R-PLK1. Mean ± SD. n.s., $P > 0.05$; *, $P < 0.05$; **, $P < 0.01$; ***, $P < 0.001$ by unpaired student's *t* test. *n* = 3 biological replicates. (**E**) Relative cell viability of mMC cells under the treatment of Vemurafenib with or without NAC for 72h. Mean ± SD. n.s., $P > 0.05$; *, $P < 0.05$; **, $P < 0.01$ by non-linear regression. (**F** and **G**) Cellular general ROS level was analyzed by CellRox under the 24h treatment of vehicle or Vemurafenib. (**F**) A375-EV versus A375-PLK1. (**G**) A375R-EV versus A375R-PLK1. Mean ± SD. n.s., $P > 0.05$; *, $P < 0.05$; **, $P < 0.01$ by unpaired student's *t* test. *n* = 3 biological replicates. (**H** and **I**) Parameters of glycolysis (**H**) and mitochondria respiration (**I**) were measured in mMC-EV and mMC-PLK1 cells respectively. Mean ± SD. n.s., $P > 0.05$; *, $P < 0.05$ by unpaired student's *t* test. *n* = 3 biological replicates. (**J**) Relative cell viability of mMPI cells under the treatment of Vemurafenib with or without 2-DG for 72h. Mean ± SD. n.s., $P > 0.05$; *, $P < 0.05$; **, $P < 0.01$ by non-linear regression. (**K**) Immunoblot of PLK1 protein level in the A375 (Top) and A375R (Bottom) after knockdown PLK1 by shRNA. (**L** and **M**) Cell viability of A375 (**L**) and A375R (**M**) under the treatment of Vemurafenib at different concentrations for 72h after PLK1 knockdown. Mean ± SD. n.s., $P > 0.05$; *, $P < 0.05$; **, $P < 0.01$ by unpaired student's *t* test. (**N**) Tumor weight (Top) and growth curve parameters (Bottom) of the allograft experiment indicated in Fig. 3**O**. Mean ± SD. n.s., $P > 0.05$; *, $P < 0.05$; **, $P < 0.01$ by unpaired student's *t*. The data underlying the graphs shown in the figure can be found in S1 Data.
