# Supplementary material for: The cell cycle regulator PLK1 promotes murine melanoma progression by regulating the transcription factor BACH1
Source: PLoS Biol. 2025 Nov 24;23(11):e3003490. doi: 10.1371/journal.pbio.3003490 (PMC12643297; doi:10.1371/journal.pbio.3003490)
Supplement: S4 Fig — (PDF) [file pbio.3003490.s004.pdf]

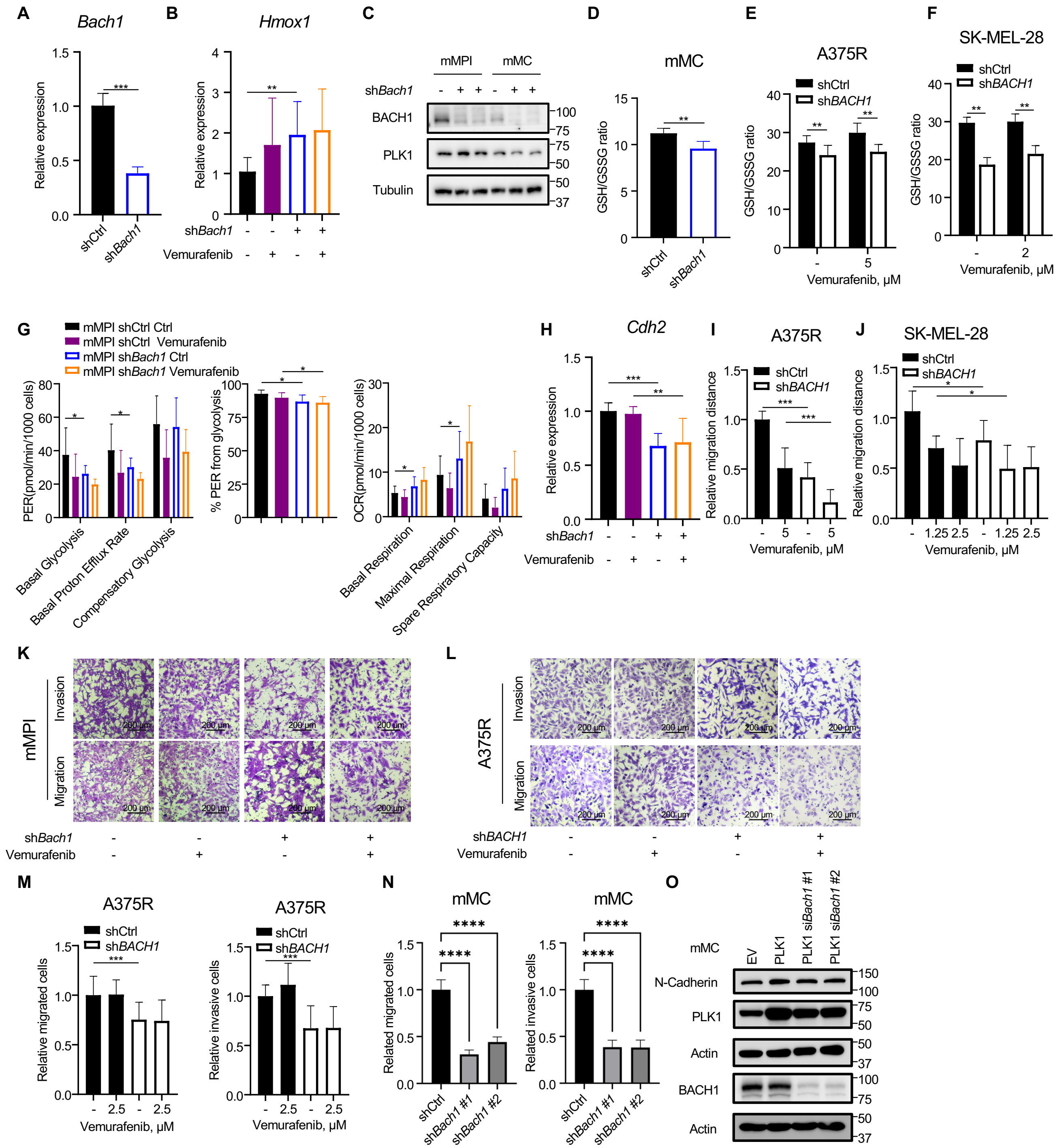

A
Bach1
Relative expression
shCtrl
shBach1
B
Hmox1
shBach1
Vemurafenib
C
mMPI
mMC
BACH1
PLK1
Tubulin
D
mMC
GSH/GSSG ratio
E
A375R
shCtrl
shBACH1
Vemurafenib, μM
F
SK-MEL-28
G
mMPI shCtrl Ctrl
mMPI shCtrl Vemurafenib
mMPI shBach1 Ctrl
mMPI shBach1 Vemurafenib
PER(pmol/min/1000 cells)
% PER from glycolysis
OCR(pmol/min/1000 cells)
Basal Glycolysis
Basal Proton Efflux Rate
Compensatory Glycolysis
Basal Respiration
Maximal Respiration
Spare Respiratory Capacity
H
Cdh2
I
Relative migration distance
J
K
Invasion
Migration
L
M
Relative migrated cells
Relative invasive cells
N
Related migrated cells
Related invasive cells
shBach1 #1
shBach1 #2
O
EV
PLK1
PLK1 siBach1 #1
PLK1 siBach1 #2
N-Cadherin
Actin

S4 Fig. BACH1 depletion reverses PLK1-associated phenotype in melanoma

(**A** and **B**) The expression of *Bach1* (**A**) and *Hmox1* (**B**) was evaluated by qRT-PCR in mMPI shCtrl and mMPI sh*Bach1* cells. Mean ± SD. n.s., *P* > 0.05; *, *P* < 0.05; **, *P* < 0.01; ***, *P* < 0.001 by unpaired student's *t*. *n* = 3 biological replicates. (**C**) Immunoblot of BACH1 and PLK1 protein level in mMPI and mMC cells after knockdown by shRNA. (**D**) GSH/GSSG ratio was detected in mouse melanoma cells mMC sh*Ctrl* and mMC sh*Bach1* cells. Mean ± SD. n.s., *P* > 0.05; *, *P* < 0.05; **, *P* < 0.01; ***, *P* < 0.001 by unpaired student's *t*. *n* = 3 biological replicates. (**E** and **F**) GSH/GSSG ratio was detected in human melanoma cells A375R (**E**) and SK-MEL-28 (**F**), with or without knockdown BACH1. Mean ± SD. n.s., *P* > 0.05; *, *P* < 0.05; **, *P* < 0.01 by unpaired student's *t*. *n* = 3 biological replicates. (**G**) Left and middle, glycolytic parameters measured by GRA in mMPI shCtrl and mMPI sh*Bach1* cells upon the treatment of vehicle or Vemurafenib. Right, measurement of mitochondria respiration parameters by Mito Stress test in mMPI shCtrl and mMPI sh*Bach1* cells. Mean ± SD. n.s., *P* > 0.05; *, *P* < 0.05 by unpaired student's *t*. *n* = 3 biological replicates. (**H**) mRNA level of *Cdh2* was measured in mMPI cells after knockdown BACH1 by shRNA. Mean ± SD. n.s., *P* > 0.05; *, *P* < 0.05; **, *P* < 0.01; ***, *P* < 0.001 by unpaired student's *t*. *n* = 3 biological replicates. (**I** and **J**) Wound healing assay of A375R (**I**) and SK-MEL-28 (**J**) with or without BACH1 knockdown. Mean ± SD. n.s., *P* > 0.05; *, *P* < 0.05; **, *P* < 0.01; ***, *P* < 0.001 by unpaired student's *t*. *n* = 3 biological replicates. (**K** and **L**) Representative images of transwell migration and invasion assay in mMPI (**K**) and A375R (**L**) cells upon the indicated treatment. Scale bar, 200 μm. (**M**) Quantification of transwell migration (Left) and invasion (Right) assay in A375R shCtrl and A375R sh*BACH1* cells upon the treatment of Vemurafenib. Mean ± SD. n.s., *P* > 0.05; *, *P* < 0.05; **, *P* < 0.01; ***, *P* < 0.001 by unpaired student's *t*. *n* = 3 biological replicates. (**N**) Quantification of transwell migration (Left) and invasion (Right) assay in mMC shCtrl and mMC sh*BACH1* cells. Mean ± SD. n.s., *P* > 0.05; *, *P* < 0.05; **, *P* < 0.01; ***, *P* < 0.001 by unpaired student's *t*. *n* = 3 biological replicates. (**O**) Immunoblots to detect protein level of BACH1 and N-Cadherin after silencing *Bach1* in mMC-PLK1 cells by siRNA. The data underlying the graphs shown in the figure can be found in S1 Data.
