# Supplementary material for: The cell cycle regulator PLK1 promotes murine melanoma progression by regulating the transcription factor BACH1
Source: PLoS Biol. 2025 Nov 24;23(11):e3003490. doi: 10.1371/journal.pbio.3003490 (PMC12643297; doi:10.1371/journal.pbio.3003490)
Supplement: S5 Fig — (PDF) [file pbio.3003490.s005.pdf]

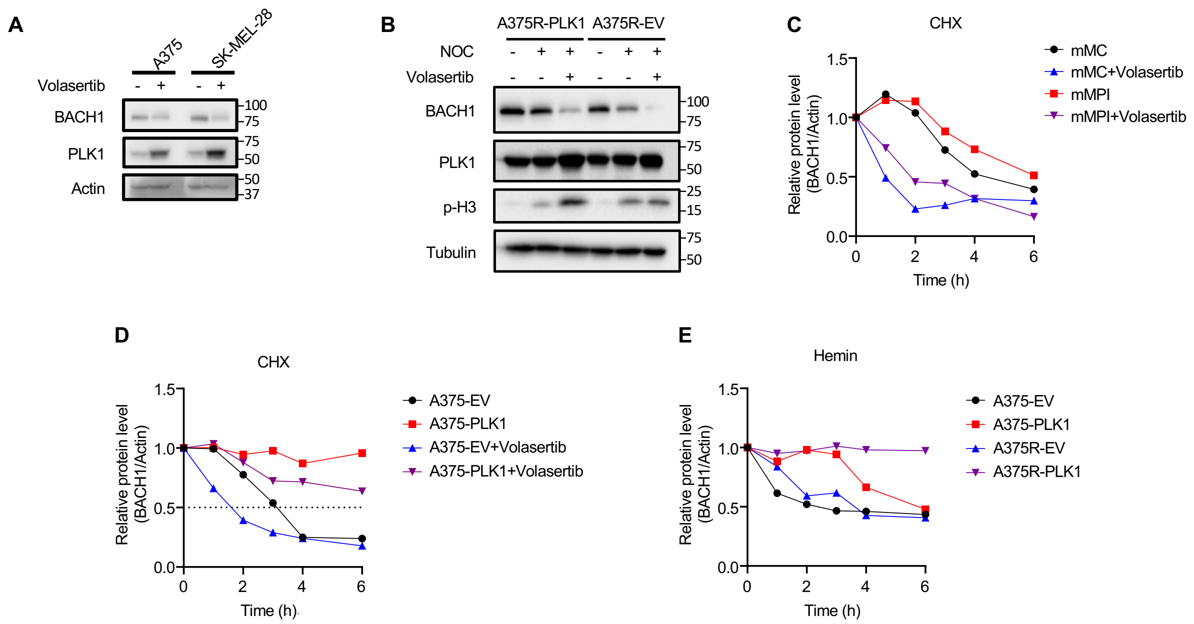

S5 Fig. BACH1 could be stabilized by PLK1

(**A**) Immunoblots of BACH1 protein level in human melanoma cells under the treatment of Vemurafenib. (**B**) Immunoblots to detect the BACH1 protein level in A375R-EV and A375R-PLK1 cells under the treatment of either DMSO, NOC, or NOC plus Volasertib. (**C-E)** Relative BACH1 protein level was quantified at each time point by immunoblots. (**C**) CHX chase in mMC and mMPI cells. (**D**) CHX chase in A375-EV and A375-PLK1 cells. (**E**) Hemin chase in A375 and A375R cells. The data underlying the graphs shown in the figure can be found in S1 Data.
