# Supplementary material for: The cell cycle regulator PLK1 promotes murine melanoma progression by regulating the transcription factor BACH1
Source: PLoS Biol. 2025 Nov 24;23(11):e3003490. doi: 10.1371/journal.pbio.3003490 (PMC12643297; doi:10.1371/journal.pbio.3003490)
Supplement: S6 Fig — (PDF) [file pbio.3003490.s006.pdf]

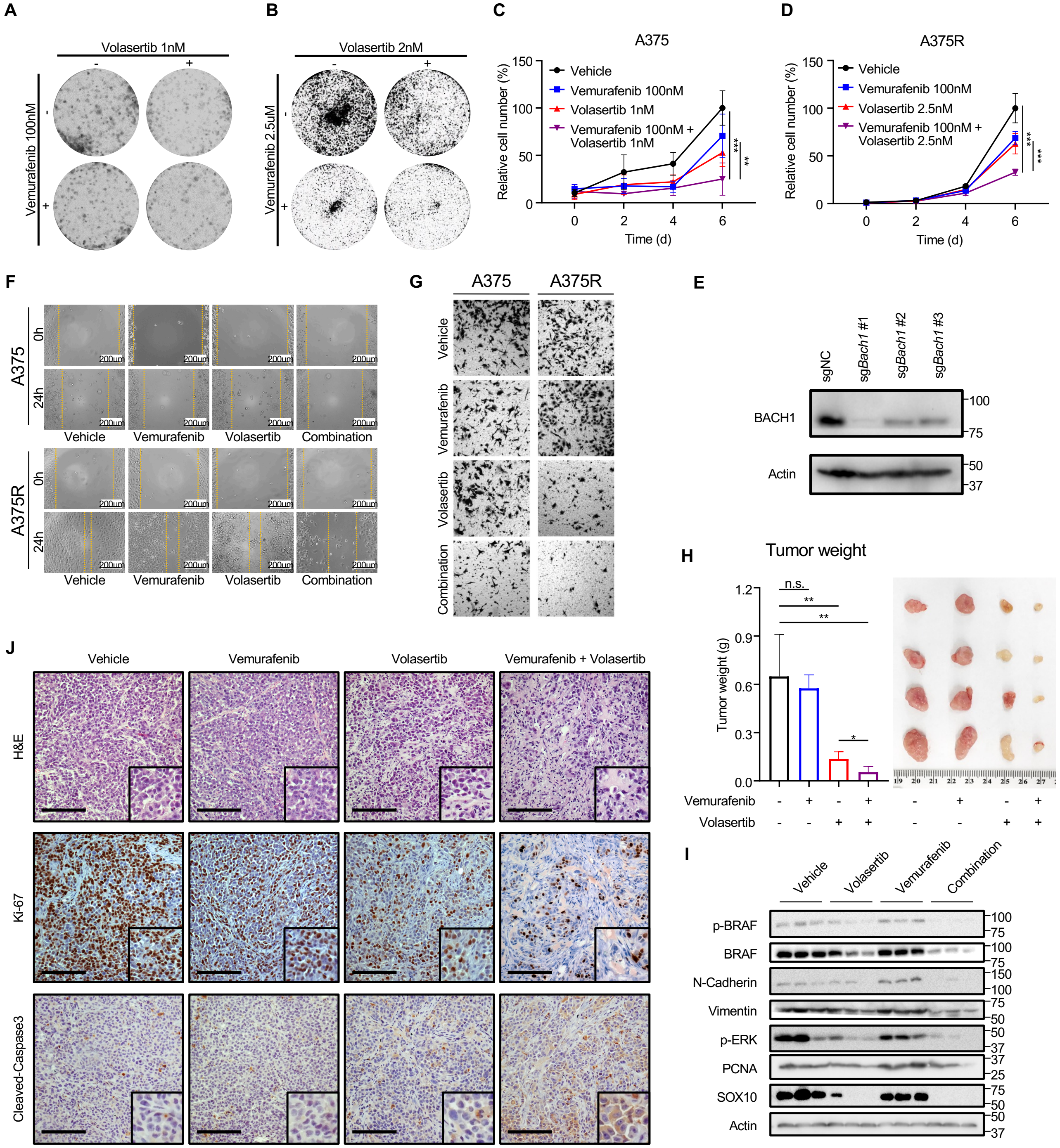

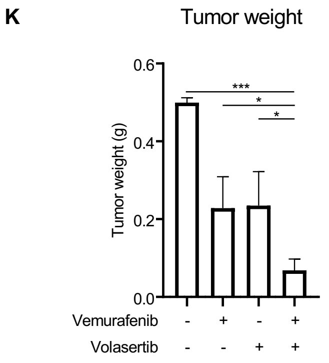

L

Exponential (Mathusian) growth	Vehicle	Volasertib	Vemurafenib	Vemurafenib +Volasertib
Y0	46.69	47.79	60.25	35.04
k	0.05511	0.04752	0.03926	0.03192
DoublingTime	12.58	14.59	17.65	21.72

S6 Fig. PLK1 inhibitor enhances the efficacy of Vemurafenib in vitro and in vivo

(**A** and **B**) Colony formation assay of A375R (**A**) and A375R (**B**) cells under the treatment of DMSO, Volasertib, Vemurafenib, or a combination of the two drugs, respectively. (**C** and **D**) Cell proliferation assay was performed by counting cell numbers in A375 (**C**) and A375R (**D**) cells upon the indicated drug treatment. Mean ± SD. n.s., $P > 0.05$; *, $P < 0.05$; **, $P < 0.01$; ***, $P < 0.001$ by unpaired student's *t* test. $n = 3$ biological replicates. (**E**) Immunoblot of BACH1 protein level in A375R BACH1-KO cells. (**F** and **G**) Representative images of wound healing assay (**F**) and transwell migration assay (**G**) performed in A375 and A375R cells under the drug treatment. Scale bar, 200 μm. (**H**) Tumors harvested from the A375R-derived xenograft. Left, weight measurement of tumors right after harvest. Mean ± SD. n.s., $P > 0.05$; *, $P < 0.05$; **, $P < 0.01$ by unpaired student's *t* test. $n = 4$ biological replicates. Right, representative images of A375R-derived tumors at the end of the study. (**I**) Immunoblots of A375R-derived tumors to detect the indicated protein expression under different treatment. (**J**) Representative images of histological staining to show the pathologic structure of A375R-derived tumors from each group. Top, H&E staining; middle, IHC staining of Ki-67; bottom, IHC staining of cleaved-Caspase 3. Scale bar, 100 μm. (**K**) Tumor weight of mMC-derived tumors was measured immediately after removal. Mean ± SD. n.s., $P > 0.05$; *, $P < 0.05$; **, $P < 0.01$; ***, $P < 0.001$ by unpaired student's *t* test. $n = 3$ biological replicates. (**L**) Parameters for growth curve of mMC-derived tumors under the different treatment conditions. The data underlying the graphs shown in the figure can be found in S1 Data.
