# Supplementary material for: The cell cycle regulator PLK1 promotes murine melanoma progression by regulating the transcription factor BACH1
Source: PLoS Biol. 2025 Nov 24;23(11):e3003490. doi: 10.1371/journal.pbio.3003490 (PMC12643297; doi:10.1371/journal.pbio.3003490)
Supplement: S3 Table — (DOCX) [file pbio.3003490.s009.docx]

| Drugs | IC_50_ | CI |
| --- | --- | --- |
| Volasertib | 11nM |  |
| Vemurafenib | 10uM |  |
| Volasertib (in combination 2.5uM Vemurafenib) | 5.5nM | CI=0.750 |

S3 Table. The IC_50_ values of Volasertib and Vemurafenib in A375R cells
