# Supplementary material for: The cell cycle regulator PLK1 promotes murine melanoma progression by regulating the transcription factor BACH1
Source: PLoS Biol. 2025 Nov 24;23(11):e3003490. doi: 10.1371/journal.pbio.3003490 (PMC12643297; doi:10.1371/journal.pbio.3003490)
Supplement: S5 Table — (DOCX) [file pbio.3003490.s011.docx]

S5 Table. Primers for qRT-PCR

| Gene | Forward | Reverse | Cat No. |
| --- | --- | --- | --- |
| *Actb* | CATTGCTGACAGGATGCAGAAGG | TGCTGGAAGGTGGACAGTGAGG | MP200232 |
| *Cdh2* | CCTCCAGAGTTTACTGCCATGAC | CCACCACTGATTCTGTATGCCG | MP201519 |
| *Vcl* | CCTATCAAGCTGTTGGCAGTAGC | TGTGGCTCCAAGCCTTCCTGAA | MP218225 |
| *Itgb2* | CTTTCCGAGAGCAACATCCAGC | GTTGCTGGAGTCGTCAGACAGT | MP206634 |
| *Ccl2* | GCTACAAGAGGATCACCAGCAG | GTCTGGACCCATTCCTTCTTGG | MP202376 |
| *Abca6* | CTGAACCTGGAAGGAGAACCAAG | TGGTGCTCACAGTCTCCTGAAC | MP201125 |
| *Aldh1l2* | GAGACTTACGCCACTGTCAACC | TCTGTCTCTGGCGTTCATCCTC | MP200512 |
| *Pparg* | GTACTGTCGGTTTCAGAAGTGCC | ATCTCCGCCAACAGCTTCTCCT | MP210761 |
| *Ndufa5* | AGAAGCCTTGCTTCAGGGTGGT | CTCTTCCACCAATGGCTCCCAT | MP208655 |
| *Ndufab1* | CATTTGTGCCGCCAGTACAGTG | GGTCCAAACTGTCTAAGCCCAG | MP208671 |
| *Timm21* | TAGTGGTGCTCTTTGGAGTCGG | TGTGTTCTGCATTTTCCTAAGGCT | MP213230 |
| *Tfam* | GAGGCAAAGGATGATTCGGCTC | CGAATCCTATCATCTTTAGCAAGC | MP221561 |
| *Twnk* | GTCTGCTGAAGGGACATCGGAA | GGCTAGTCTCACGTTGCTGATC | MP211716 |
| *Atp5d* | ACTGGAGCCTTTGGCATCTTGG | AGTCGGCATTCACAGTGACGGA | MP201037 |
| *Atp5g1* | CATCGACACAGCAGCCAAGTTC | CCAGAATGGCATAGGAGAAGAGC | MP201028 |
| *Atp5j* | TTGAAGAGGAACATTGGTGTTACAG | GCCAATATCAACAGGTCCTCCAG | MP201032 |
| *Ndufs7* | GGCTGAGTATGTGGTGACCAAG | AGCCATGTGCATCATCTCCACG | MP208680 |
| *Bach1* | CCATGACATCCGCAGAAGGAGT | GCGTTGACAGAATGTGGTCTCG | MP201472 |
| *Hmox1* | CACTCTGGAGATGACACCTGAG | GTGTTCCTCTGTCAGCATCACC | MP206003 |
| *Timm8b* | GCATCACTTCATGGAACTATGTTG | CGGTGATGGCAAGAGTAGTGTC | MP217247 |
| Human *GAPDH* | GGAGCGAGATCCCTCCAAAAT | GGCTGTTGTCATACTTCTCATGG |  |
| Human *HK2* | GAGCCACCACTCACCCTACT | CCAGGCATTCGGCAATGTG |  |
| Mouse *Gapdh* | AGGTCGGTGTGAACGGATTTG | GGGGTCGTTGATGGCAACA |  |
| Mouse *Hk2* | ATGATCGCCTGCTTATTCACG | CGCCTAGAAATCTCCAGAAGGG |  |
