# Supplementary material for: The cell cycle regulator PLK1 promotes murine melanoma progression by regulating the transcription factor BACH1
Source: PLoS Biol. 2025 Nov 24;23(11):e3003490. doi: 10.1371/journal.pbio.3003490 (PMC12643297; doi:10.1371/journal.pbio.3003490)
Supplement: S6 Table — (DOCX) [file pbio.3003490.s012.docx]

| Gene | Forward | Reverse |
| --- | --- | --- |
| Human *GAPDH* | CTGCCCTCCTACCAGAAGAA | GGATATTGAGGGCAGGGTGA |
| Human *HK2* | AGAGGCCCGTTTTTCCAGTC | CTGAGATGGGACGTGTGGTC |
| Mouse *Gapdh* | CTCAACTTTTCCGCAGCCTT | GGGAGAACAGGGGAAATGGA |
| Mouse *Hk2* | TGAGTTACCTGTGTGCGGAT | AAGGCTCTGCTCCACTTTCT |

S6 Table. Primers for ChIP-qRT
