# Supplementary material for: The cell cycle regulator PLK1 promotes murine melanoma progression by regulating the transcription factor BACH1
Source: PLoS Biol. 2025 Nov 24;23(11):e3003490. doi: 10.1371/journal.pbio.3003490 (PMC12643297; doi:10.1371/journal.pbio.3003490)

Figure 1D

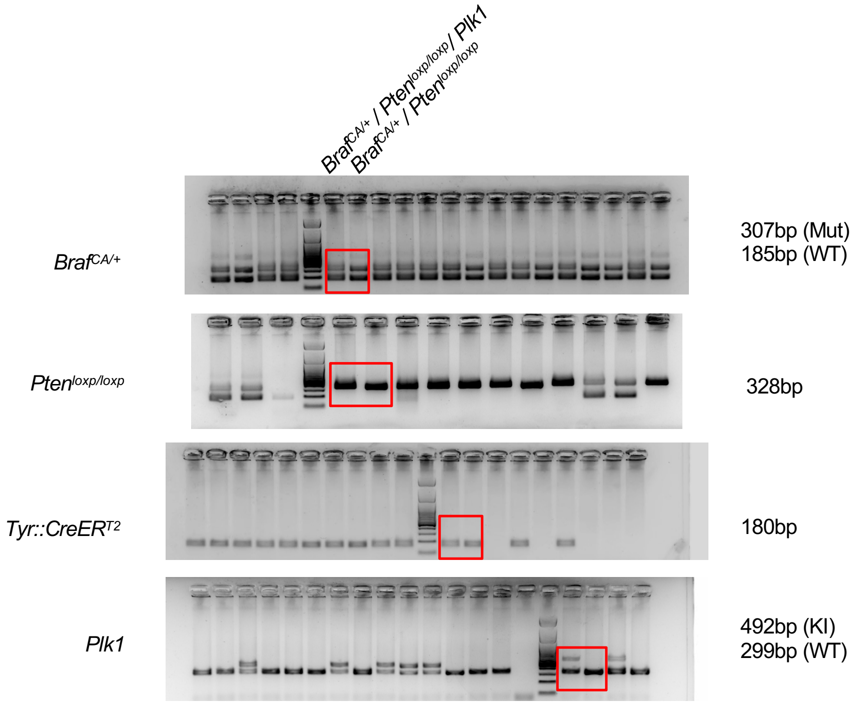

Braf^CA/+ / Pten^loxp/loxp / Plk1
Braf^CA/+ / Pten^loxp/loxp
Braf^CA/+
307bp (Mut)
185bp (WT)
Pten^loxp/loxp
328bp
Tyr::CreER^T2
180bp
Plk1
492bp (KI)
299bp (WT)

Fig 3C

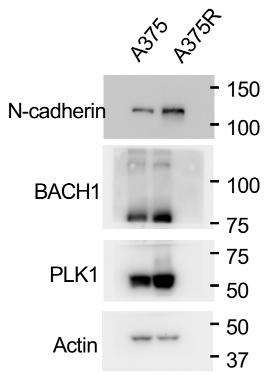

Fig 3F

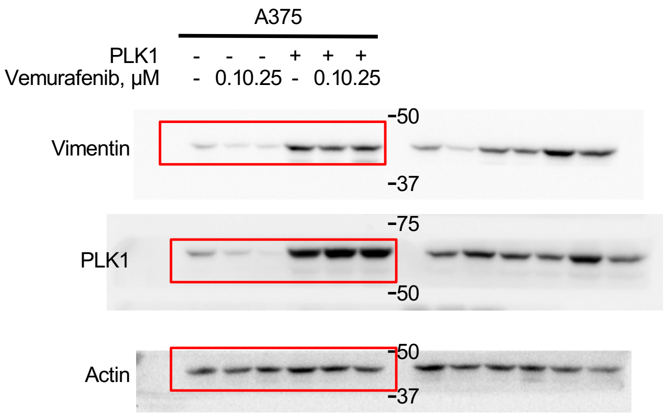

Fig 3G

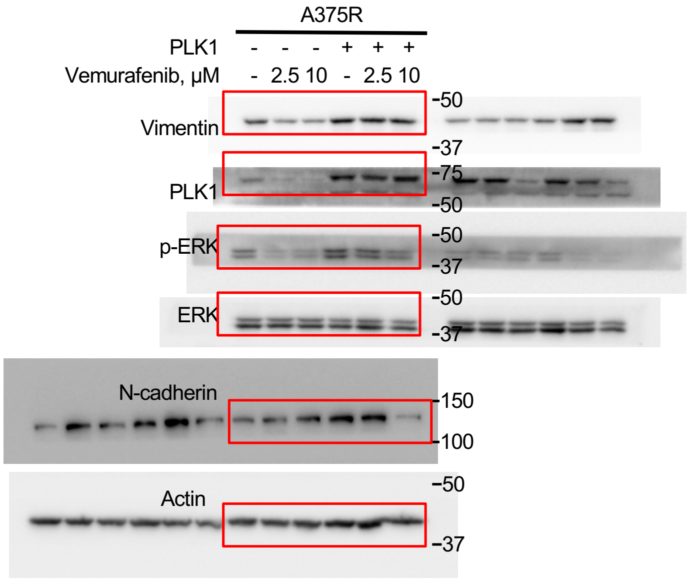

Fig 3Q

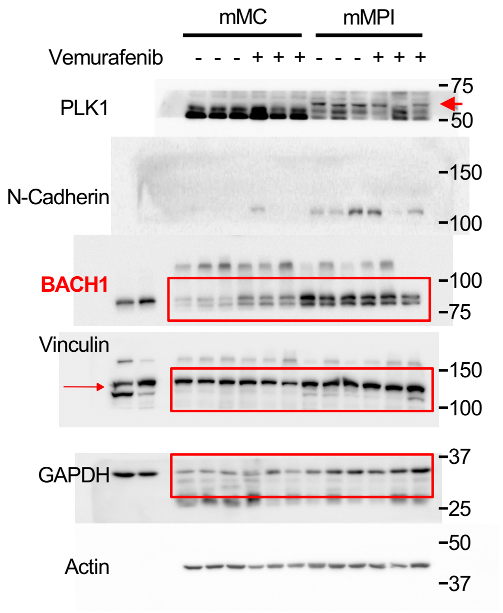

Fig 4A

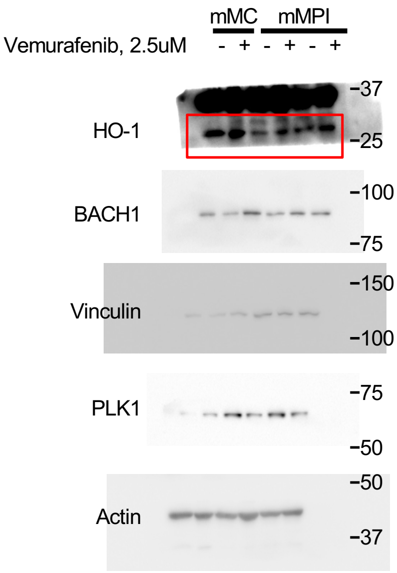

Fig 4B

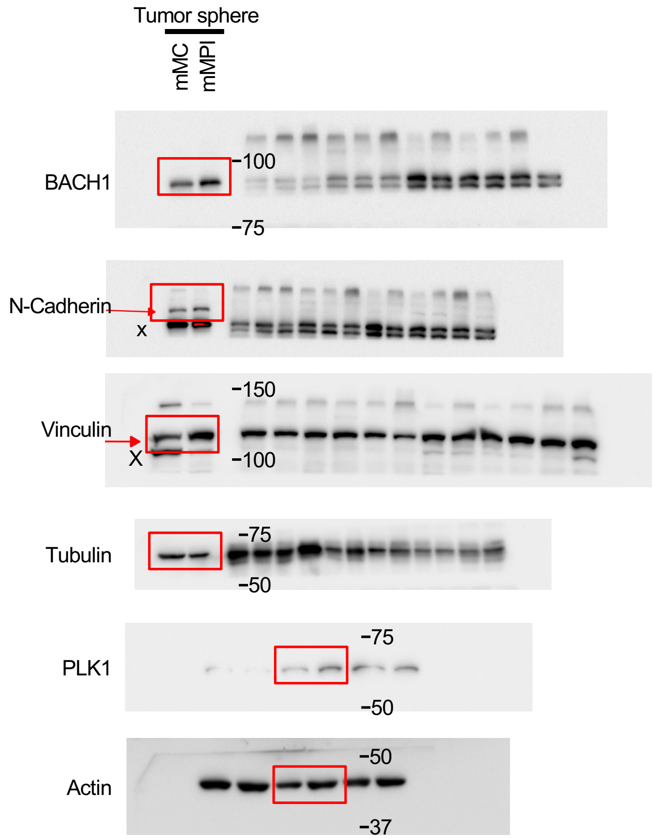

Fig 4C

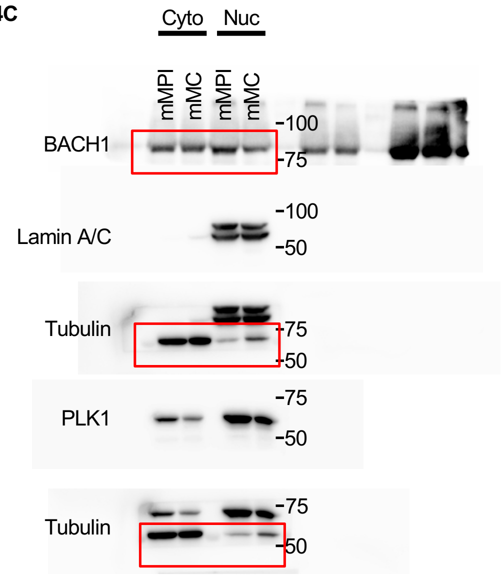

Fig 5A

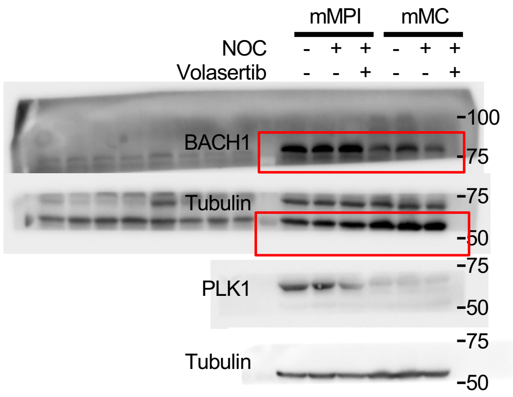

Fig 5B

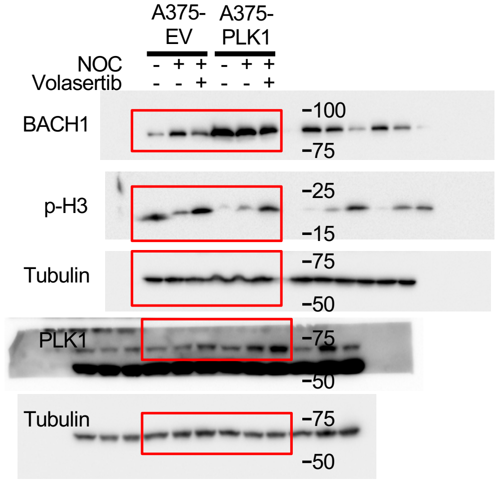

Fig 5C

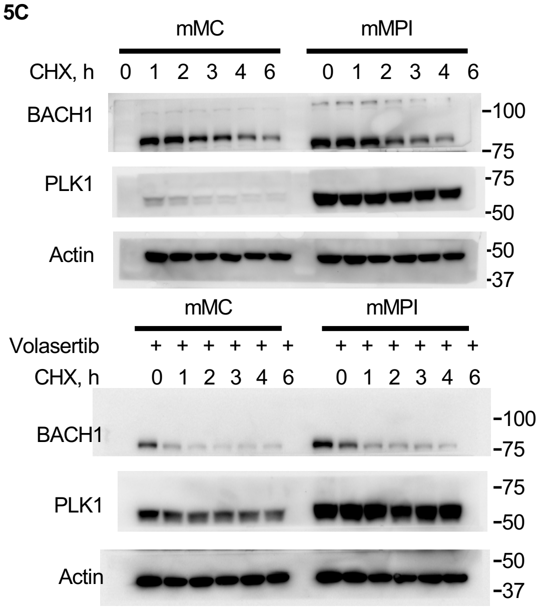

Fig 5D

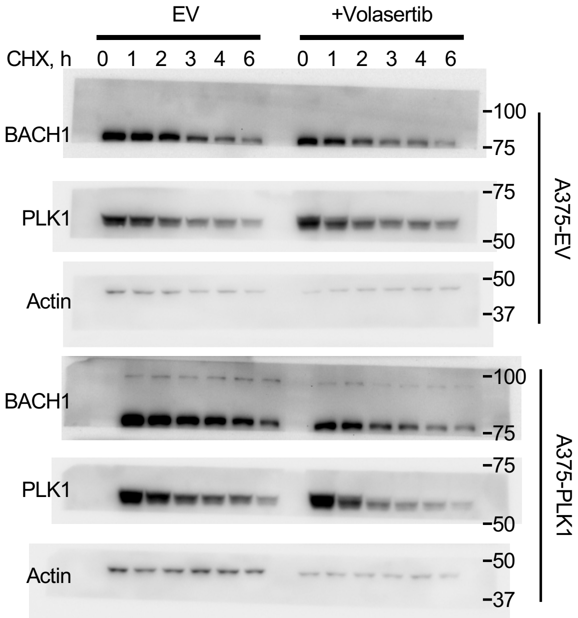

Fig 5E

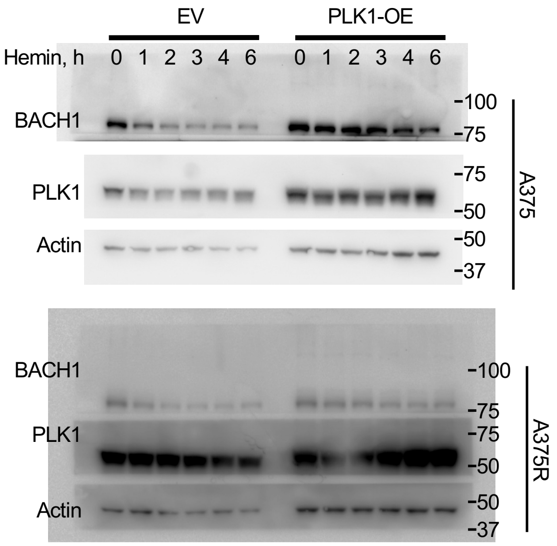

Fig 5F

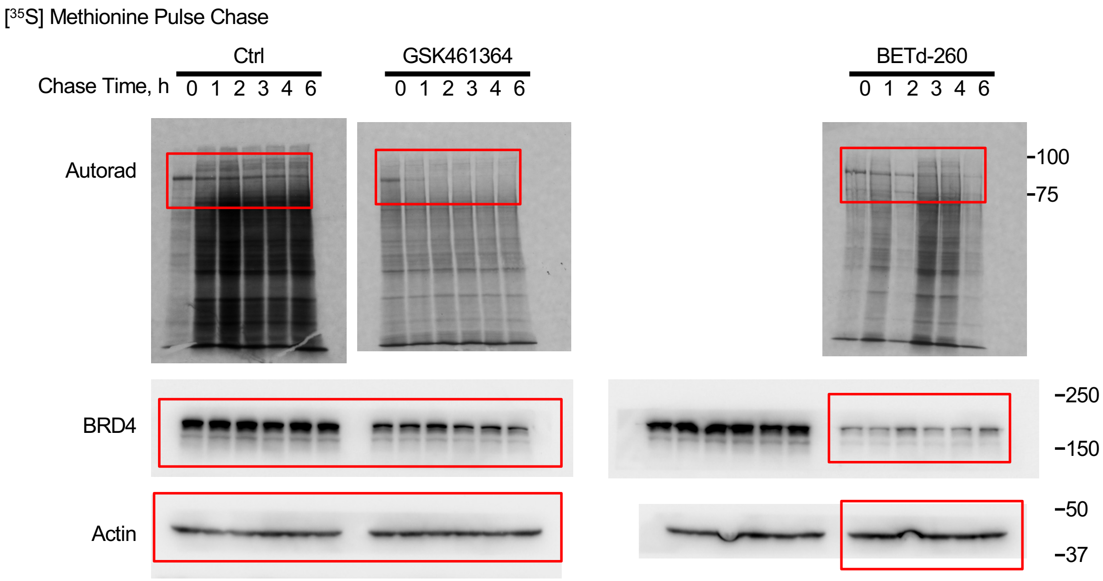

[35S] Methionine Pulse Chase
Ctrl
GSK461364
BETd-260
Chase Time, h
0 1 2 3 4 6
0 1 2 3 4 6
0 1 2 3 4 6
Autorad
BRD4
Actin
100
75
250
150
50
37

Fig 6A

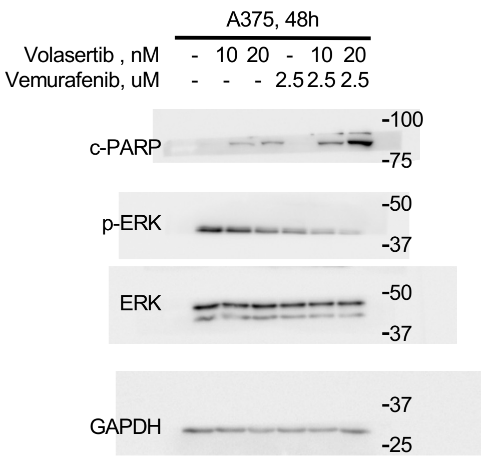
A375, 48h
Volasertib , nM - 10 20 - 10 20
Vemurafenib, uM - - - 2.5 2.5 2.5
c-PARP
-100
-75
p-ERK
-50
-37
ERK
-50
-37
GAPDH
-37
-25

Fig 6B

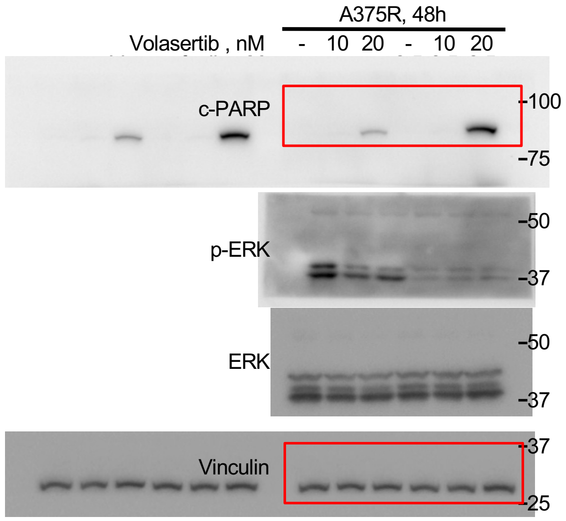
A375R, 48h
Volasertib , nM - 10 20 - 10 20
c-PARP
-100
-75
p-ERK
-50
-37
ERK
-50
-37
Vinculin
-37
-25

Supplementary Figure 1C

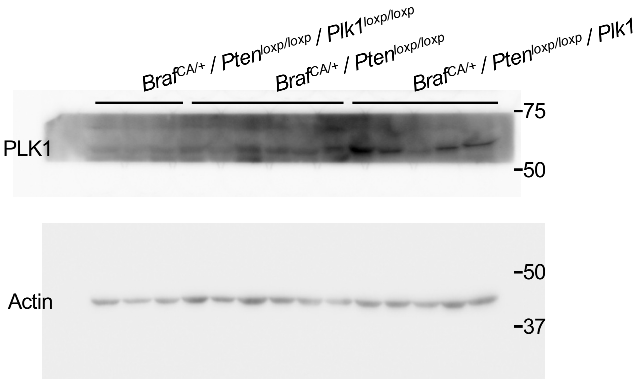

Supplementary Figure 1I

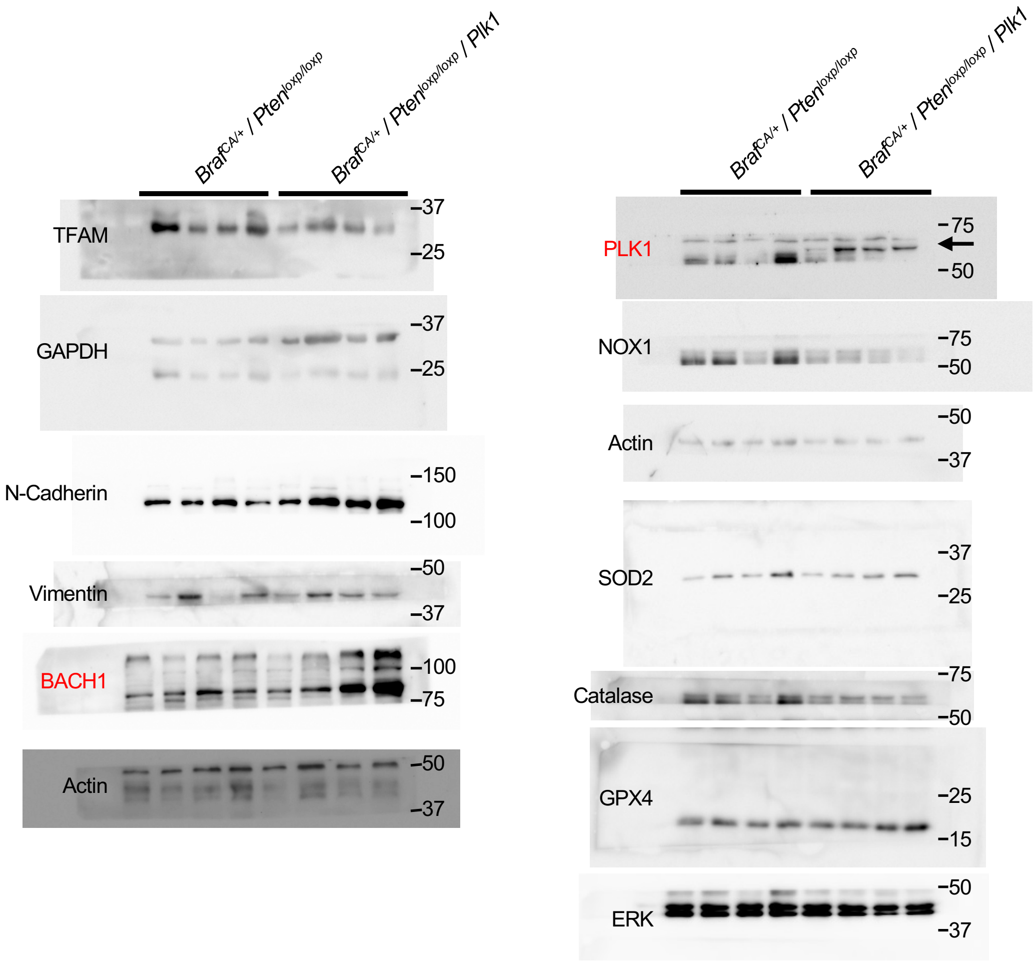

Supplementary Figure 2A

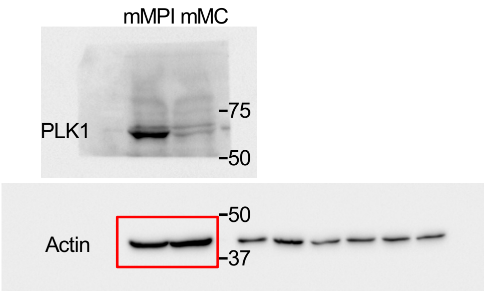

Supplementary Figure 2E

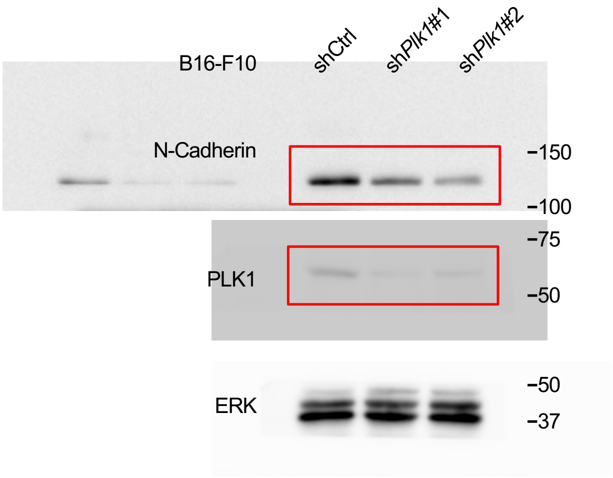

Supplementary Figure 2N

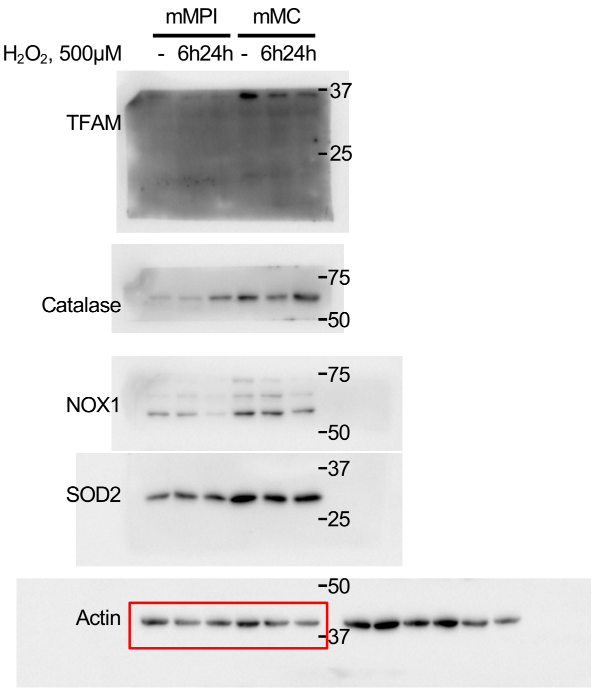

Supplementary Figure 3K

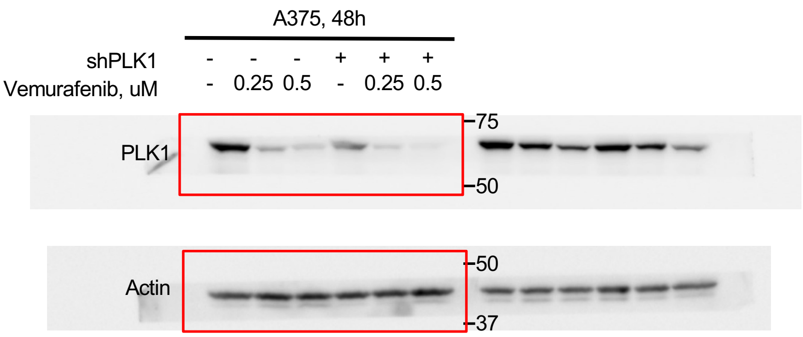

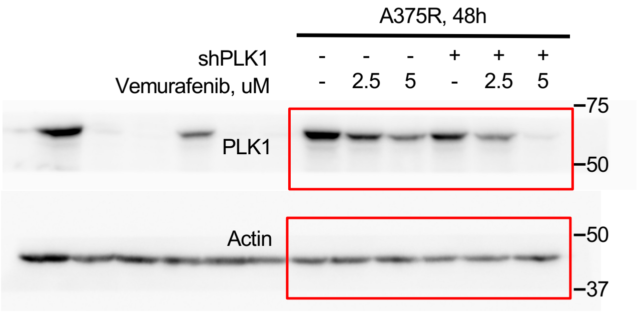

Supplementary Figure 4C

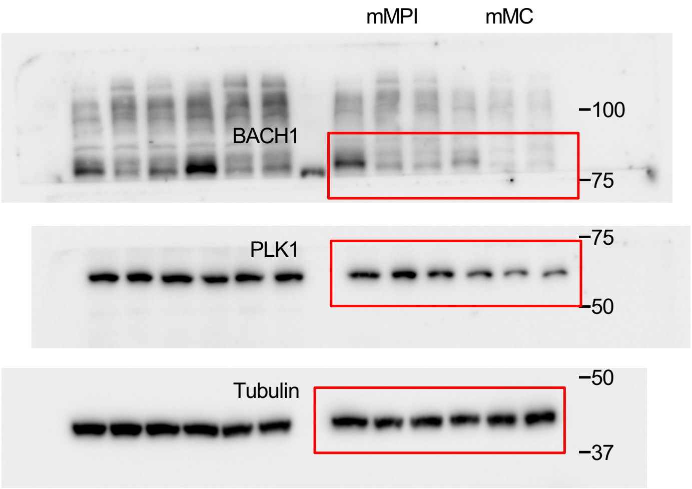

Supplementary Figure 4O

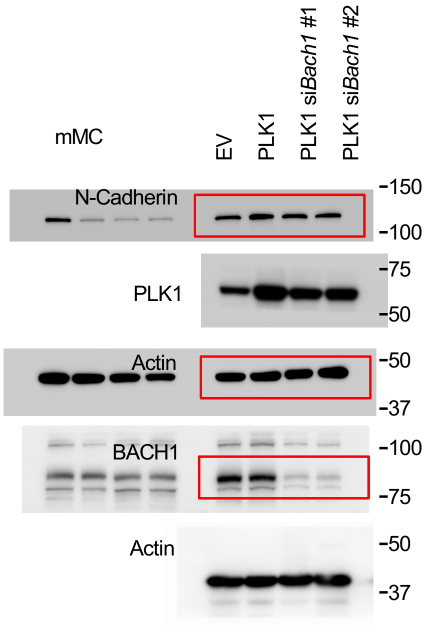

Supplementary Figure 5A

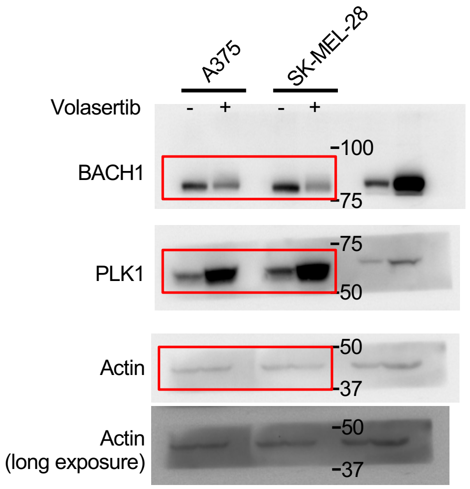

Supplementary Figure 5B

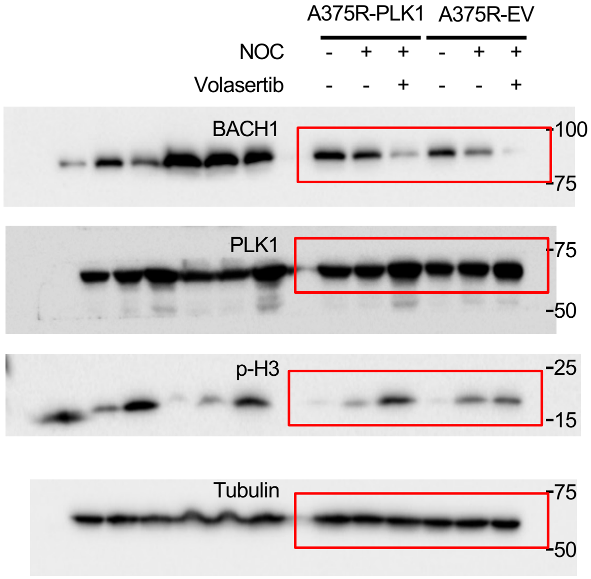

Supplementary Figure 6E

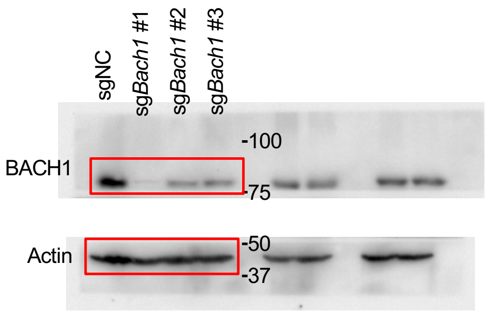

Supplementary Figure 6I

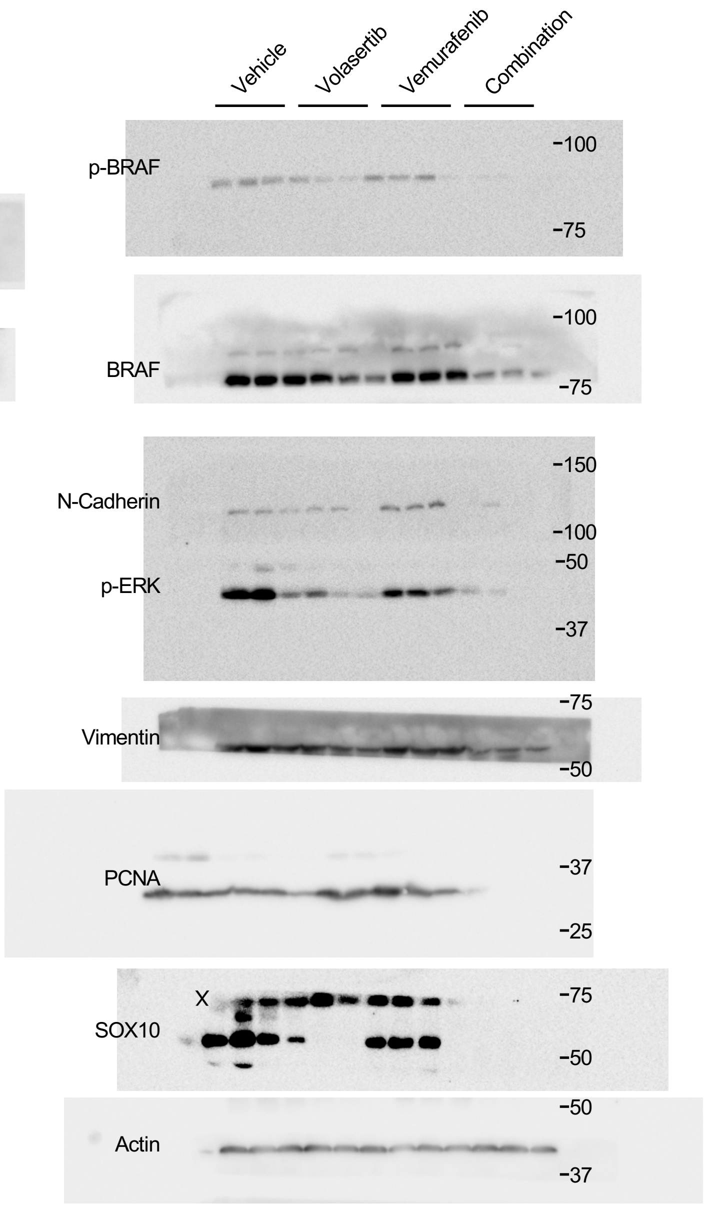

Supplement: S1 Raw image — (PDF) [file pbio.3003490.s013.pdf]
